# Psychotherapy and Teletherapy in U.S. Latinx Populations: A Narrative Review

**DOI:** 10.7759/cureus.116068

**Published:** 2026-09-10

**Authors:** Sofía I Uranga, Seneca Magallanes, Syed Wahid, Sayana Isaac, Zaheer R Munshi, Miguel R Sanchez, Alejandra E Morfin Rodriguez, John L Havlik

**Affiliations:** 1 Biological Sciences, The University of Chicago, Chicago, USA; 2 Psychiatry and Behavioral Sciences, Stanford University, Stanford, USA

**Keywords:** cultural mental health, latin american patients, mental health services, psychiatry and mental health, telepsychiatry

## Abstract

Latinx patients possess a rich cultural background and shared heritage; they also possess culturally distinct medical beliefs and practices, including beliefs around mental health and interactions with mental health systems. Most forms of psychotherapy were not developed for Latinx populations, and the efficacy of existing and novel forms of psychotherapy in Latinx patients as a group has received precious little study. In this study, we review psychotherapy and telepsychotherapy interventions for Latinx patients with mental health disorders. We conducted a literature review applying Preferred Reporting Items for Systematic Reviews and Meta-Analyses (PRISMA) guidelines, using search criteria involving terms such as “Hispanic,” “Latinx,” “psychotherapy,” and “telepsychiatry.” We selected peer-reviewed articles on psychotherapeutic interventions within U.S.-based Latinx populations and excluded studies that summarized existing findings, had limited generalizability, or involved treatments outside the study's scope. Using PubMed, Web of Science, Embase, Cochrane Library, and MEDLINE Ovid databases, we identified 45 articles for our final review. We examined the efficacy of these treatments across depressive, anxious, and other psychiatric disorders in U.S. Hispanic participants to establish both areas of strength in current approaches and areas of ongoing need for improvement. Evidence surrounding therapy and teletherapy in Latinx patients has focused on reducing the burdens of depression, anxiety, and substance use. Culturally adapted behavioral therapies, delivered both in-person and remotely, are the most studied forms of therapy in these populations. Comparative effectiveness studies have been limited, with many studies reporting simple pre-post differences and differences versus status quo or waitlist controls. Limited evidence shows that teletherapy can expand access for rural and limited English proficiency groups and be cost-effective compared with in-person care. The current evidence base around psychotherapy in Latinx patients supports culturally adapted cognitive behavioral therapy (CBT)/behavioral therapies, delivered in-person and via telehealth, as effective, acceptable, and often cost-effective for Latinx populations across depression, anxiety/post-traumatic stress disorder (PTSD), eating disorders, and substance use. Other forms of psychotherapy have also shown promise but are less well studied. Across conditions and interventions, family involvement and targeted cultural adaptations appear to improve both engagement and outcomes.

## Introduction and background

Latinx people comprise over 20% of the total U.S. population, constituting over 68 million individuals and growing [1]. This makes the Latinx population the largest ethnic minority group in the United States [2]. While incredibly heterogeneous in race, language, national origin, socioeconomic status, and immigration experiences, Latinx communities share common cultural threads [3,4]. These threads include familism (which emphasizes strong family bonds) and collectivism, as well as religious affiliations, relatively strong connections to extended social networks, and a mixed indigenous and colonial heritage [3,4]. These cultural values profoundly impact perceptions of mental health, help-seeking behaviors for mental illness, and therapeutic engagement with mental healthcare providers [5]. The unique cultural threads inherent in the U.S. Latinx experience shape care delivery from symptoms at presentation to the severity of illness at which Latinx patients access psychiatry- or psychology-based mental health services; indeed, many Latinx individuals prefer to seek support from family members or religious leaders rather than the relatively medicalized forms of care offered by psychiatrists and other licensed and credentialed mental healthcare practitioners [6].

Beyond unique stated and enacted preferences for how their mental healthcare is delivered, Latinx individuals face substantial difficulties when trying to access psychiatric and psychological services [5]. In 2024, Hispanic/Latino adults were 28% less likely than U.S. adults to have received mental health treatment in the past year, even after controlling for mental illness prevalence - only 36.1% of Latino adults with a mental illness received related services, compared to 52.4% of white adults [7]. These disparities in treatment stem from cultural and structural factors. Cultural factors include stigma surrounding mental illness (embodied in sayings such as "la ropa sucia se lava en casa" or don't air your dirty laundry in public), fears of being labeled "locos" (crazy), gender role expectations rooted in machismo, and reluctance to discuss personal struggles outside the family [5,8]. Structural barriers are equally formidable. Higher poverty rates, language barriers for those with limited English proficiency, high costs and lack of insurance, the longstanding shortage of bilingual and bicultural mental health professionals, and limited culturally relevant services all contribute to inadequate care access [9]. According to 2022 American Psychological Association data, Latinx individuals comprise just 8% of the psychology workforce despite representing nearly one-fifth of the U.S. population [10]. For undocumented immigrant Latinx populations, fear of deportation, complex acculturation stressors, and trauma exposure during migration compound these barriers [11,12]. Language barriers between Spanish-speaking Latinx patients and their predominantly non-Latinx clinician counterparts present a substantial barrier in mental health, where nuanced emotional understanding is critical [5]. Providers who do speak Spanish may still exhibit a lack of cultural competence, which further exacerbates disparities, as clinicians may misinterpret culturally bound expressions of distress such as "nervios," which can culturally refer to particular presentations of a nervous breakdown but literally translate to “nerves,” or misunderstand somatization of mental health symptoms [5,13].

Unfortunately, inquiry into how to best overcome these barriers to mental healthcare for Latinx patients has been fairly limited. A key form of care for mental illness is psychotherapy, a dialogue-based treatment with varying styles in which a provider collaboratively addresses behaviors and challenges with patients [14]. A critical methodological issue plaguing the existing psychotherapy research literature for Latinx populations is that racial/ethnic analysis of psychotherapeutic efficacy is often treated as a subgroup analysis “box-checking” footnote, which limits important and more nuanced questions of efficacy [14]. Even many studies claiming to examine interventions for "Latinx patients" actually study majority-Latinx samples without requiring participants to meet rigorous thresholds of ethnic composition that would justify population-specific conclusions. Reviews of research in cognitive behavioral modalities found a corresponding low level of research focusing on Latinx patients, with these levels being even lower for Spanish-speaking Latinx [14]. Studies conducted purely with Spanish-speaking populations in other countries, without attention to the U.S. context, fail to capture the unique experiences of U.S.-based Latinx communities (including immigration-related trauma, acculturation stress, discrimination, navigating between two cultures, and the specific structural barriers embedded in American healthcare systems) [15]. The result is a body of research literature that inadequately represents the efficacy of mental health interventions for the U.S. Latinx population, an area of research made increasingly important by the recent rapid shift of psychotherapeutic modalities to telehealth [16].

Beyond socially isolating the U.S. populace, which inhibits cultural connection, the COVID-19 pandemic catalyzed rapid expansion of telehealth services in psychiatric care. The percentage of mental health treatment facilities offering these services more than doubled from 39.4% in 2019 to 88.1% in 2022 [16]. While telehealth services show great promise to increase access to care (particularly for rural, underinsured, and limited English proficiency populations), these digital health advances may be leaving Latinx patients behind [17]. Certain groups, including the poor, the less educated, and minorities, particularly African American and Hispanic communities, have historically been left behind in being able to use digital services for their benefit [17]. Access to broadband internet, digital literacy, comfort with technology, and availability of culturally adapted telehealth platforms are all factors that likely impact receipt of mental healthcare for Latinx communities [18]. Understanding how telepsychotherapy, psychotherapy in a remote medium such as by video call, functions for Latinx populations specifically, including both its promise and its pitfalls, is essential for improving mental healthcare delivery to this unfortunately underserved and often vulnerable group of patients.

Accordingly, this study sought to review the literature on psychotherapy and telepsychotherapy interventions specifically designed for, or rigorously tested in, U.S.-based Latinx populations. This review synthesized studies and subanalyses of psychotherapy and telepsychotherapy interventions for mental illness in ≥95% of U.S. Latinx populations, examining therapy efficacy across depressive, anxiety, substance use, and other psychiatric disorders. By cataloging evidence-based treatments and efficacy data across both in-person and telehealth interventions, this review delineates both areas of strength in current approaches and areas of critical need for future research and clinical innovation to serve Latinx patients more effectively.

## Review

Methodology

This narrative review without synthesis followed the synthesis without meta-analysis (SWiM) reporting guidelines. On November 24, 2023, we conducted a search for studies of psychotherapy and telepsychotherapy interventions for Latinx patients published from 1946 to our search date using the PubMed, Web of Science, Embase, Cochrane Library, and MEDLINE Ovid databases. We conducted a literature search according to Preferred Reporting Items for Systematic Reviews and Meta-Analyses (PRISMA) guidelines. Searches for the study involved a combination of terms such as “Hispanic,” “Latinx,” “psychotherapy,” and “telepsychiatry.” We used “OR” search logic to capture all possible studies of interest. The full search criteria, logic, and keywords used are outlined in the appendix. Importantly, the search was limited to studies published in peer-reviewed journals written in English. Given the substantial variety in study type, intervention models, and targeted subpopulations, we performed a thorough search for peer-reviewed studies applying systematic review methods but presenting our findings as a narrative review.

Studies included in our review were limited to (1) English-language peer-reviewed articles about interventional studies (including retrospective analyses, randomized and unrandomized controlled trials, interventional studies, feasibility studies, comparative effectiveness studies, and clinical efficacy studies), which (2) reported efficacy of psychotherapeutic interventions within populations that were reported as 95% or greater Hispanic/Latinx/Latino/Latina/Latine, whether across the entire study or in explicit subpopulation analysis. We excluded review articles, study protocols, case studies, and letters to the editor, as these forms of research either summarized existing advances in the field or had limited generalizability. We further excluded treatments outside the scope of study focus (e.g., deep brain stimulation) and articles with participants not diagnosed with a Diagnostic and Statistical Manual of Mental Disorders (DSM) mental health disorder. Finally, we excluded studies on patients not based in the United States. Our search strategy identified 1,488 unique records, of which 1,355 were excluded based on non-automated, independent abstract review by three researchers (JLH, SU, SM), as detailed in the PRISMA flow diagram in Figure 1. Each of the 131 retrieved full-text articles was then independently reviewed by the same three researchers (JLH, SU, SM) without automation and subject to the inclusion and exclusion criteria above, which yielded 45 articles included in our final review. Data were extracted independently by SU and SM using a data extraction table that included the first author, study title, disorder described in the study (e.g., depression), study type (e.g., clinical efficacy), publication year, data source (e.g., RCT), intervention setting, number of participants (n), study design, main outcome (e.g., PTSD symptoms), measurement instrument (e.g., Patient Health Questionnaire-9 {PHQ-9}), and main findings. Risk of bias analyses were not performed on the final 45 articles. The authors resolved all disagreements on study inclusion and data collection by consensus.

**Figure 1:**
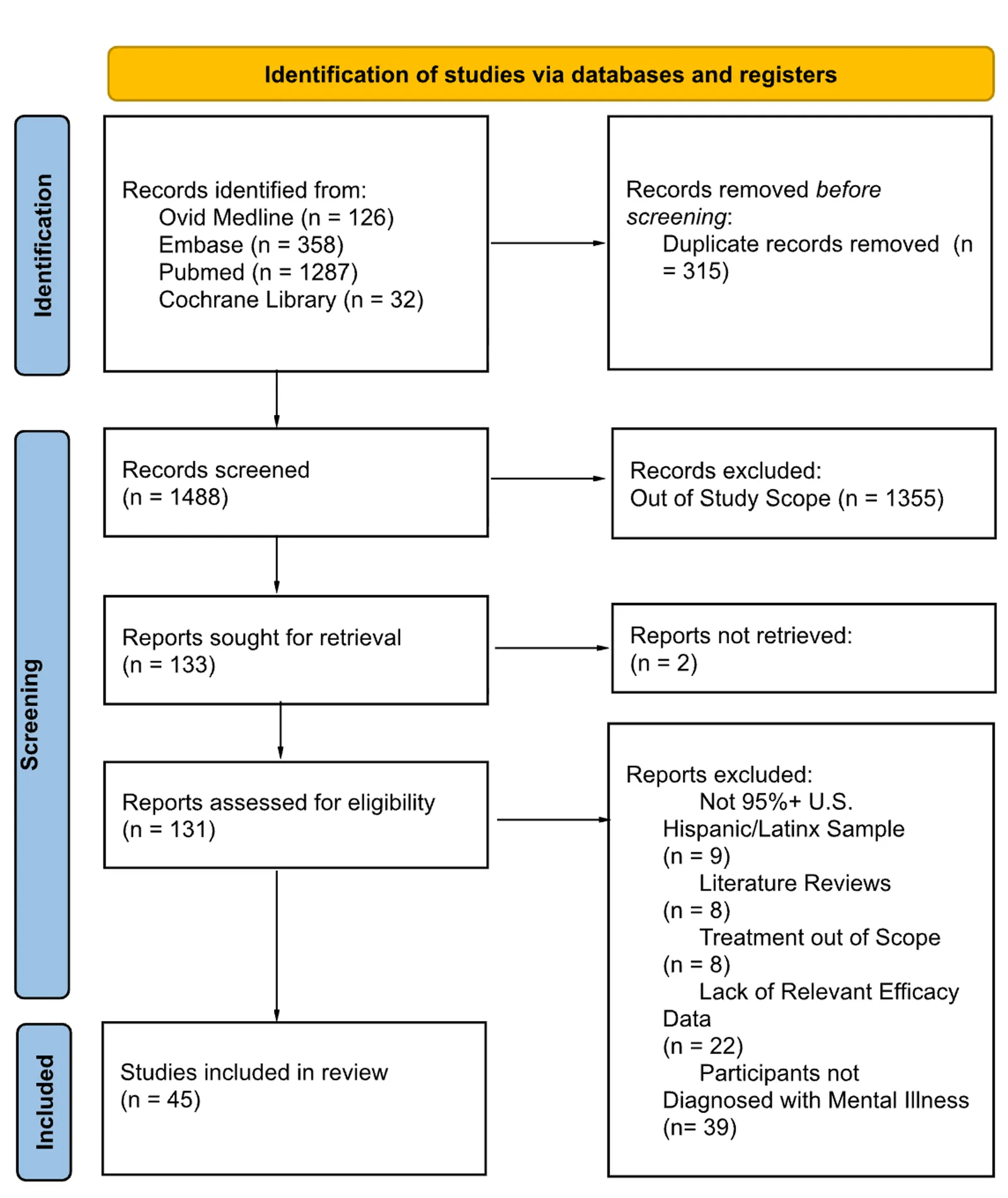
PRISMA flow diagram of the study selection process.

Results

Part 1: Therapeutic Interventions to Treat Depression in Hispanic/Latinx U.S. Populations

Conventional therapy interventions: Latinx patients with anxiety and depression have been a focus of several notable trials of new, culturally adapted, and established forms of psychotherapy (Table 1). Ell et al. published a randomized controlled trial of a collaborative care model designed to expose patients with diabetes and comorbid depression to depression psychotherapy and/or pharmacotherapy [19]. The study consisted of 387 participants, of whom 96.5% (n=372) were Hispanic. The participants were randomized to an enhanced usual care (EUC) group and an intervention group (INT). The INT group received problem-solving therapy, a practically oriented form of therapy focused on improving coping and problem-solving skills, and/or medication management based on a stepped-care algorithm of first-line treatment, telephone treatment, adherence, relapse prevention, and systems navigation assistance. INT patients were significantly more likely to have an equal to or greater than 50% reduction in the Depression Symptom Checklist (SCL-20) score than EUC patients. Specifically, the INT group had 57%, 62%, and 62% reductions at six, 12, and 18 months, respectively, while the EUC group experienced 36%, 42%, and 44% at these same time points; INT patients had significantly higher odds of ≥50% reduction in symptoms (odds ratio: 2.46-2.57; p<0.001). This randomized controlled trial offered strong evidence for the authors’ claim that socioculturally tailored collaborative care can improve depression symptoms in a predominantly Hispanic patient population with diabetes.

**Table 1:** Psychotherapy trials for major depressive disorder in Hispanic/Latinx U.S. populations. CCAPS-34: Counseling Center Assessment of Psychological Symptom; HSI: Hispanic-serving institutions; PWI: predominantly White institutions; MADRS: Montgomery-Åsberg Depression Rating Scale; Q-LES-Q: Quality of Life Enjoyment and Satisfaction Questionnaire; PHQ-9: Patient Health Questionnaire-9; SDS: Sheehan Disability Scale; SF-12: MOS Short-Form Health Survey; SCL-20: 20-item Symptom Checklist; HSCL-20: 20-item Hopkins Symptom Checklist; BDI-II: Beck Depression Inventory-II; CDI: Children's Depression Inventory; CDRS-R: Children's Depression Rating Scale-Revised; SIQ-Jr: Suicidal Ideation Questionnaire-Junior; CBT: cognitive behavioral therapy; TEPSI: Talleres de Educacion Psicologica; BADS: Behavioral Activation for Depression Scale; TAC: Therapeutic Alliance with Clinician Questionnaire; BATD: behavioral activation treatment for depression; WI: Wisconsin; HRSD: Hamilton Rating Scale for Depression; DBRS: Depressive Behavior Rating Scale; HSCL: Hopkins Symptom Checklist; SCL: Symptom Checklist; BAL: behavioral activation for Latinos; PTSD: post-traumatic stress disorder; CPSS: Child PTSD Symptom Scale; CDI: Child Depressive Index; CDRS-R: Children’s Depression Rating Scale - Revised; CGI: Clinical Global Impression scale; CGAS: Global Assessment Scale for Children; SAS-SR: Social Adjustment Scale - Self-Report; IPT-A: interpersonal psychotherapy for depressed adolescents; PST-PC: problem-solving treatment for primary care; PSWQ: Penn State Worry Questionnaire; ECLA: Engagement and Counseling for Latinos; MA: Massachusetts; PR: Puerto Rico; LA: Los Angeles; CA: California; NY: New York; TX: Texas

Studies	Study title	Study type	Publication year	Data source	Setting	Study design	Study, n	Main outcome	Measure instrument	Main findings
Ell et al. [19]	Collaborative care management of major depression among low-income, predominantly Hispanic subjects with diabetes: a randomized controlled trial	RCT	2010	RCT	Community safety-net clinics in CA	RCT	387	Depressive severity ratings	SCL-20, PHQ-9	Socioculturally and organizationally adapted collaborative care is feasible and effective in improving quality of depression treatment and depressive and functional outcomes in a low-income, predominantly Hispanic population in safety-net clinics
Ell et al. [20]	One-year post-collaborative depression care trial outcomes among predominantly Hispanic diabetes safety-net patients	RCT	2011	RCT	Community safety-net clinics in CA	RCT	264	Depression severity ratings	SCL-20, SF-12, PHQ-9	Socioculturally tailored collaborative care that included maintenance antidepressant medication, ongoing symptom monitoring, and behavioral activation relapse prevention was associated with depression improvement over 24 months for predominantly Hispanic patients in primary safety-net care
Hay et al. [21]	Cost-effectiveness analysis of collaborative care management of major depression among low-income, predominantly Hispanics with diabetes	RCT	2011	RCT	LA County public community clinics	RCT	387	Cost-effectiveness and depression free days	MOS SF-12, PHQ-9	Socioculturally adapted collaborative depression care improved utility and quality of life in predominantly low-income Hispanic diabetes patients and was highly cost-effective
Heilemann et al. [22]	Schema therapy, motivational interviewing, and collaborative-mapping as treatment for depression among low-income, second-generation Latinas	Controlled clinical trial	2011	Controlled clinical trial	CA	Controlled clinical trial	8	Depression severity ratings	BDI-II, Resilience Scale	Customized intervention of schema therapy and motivational interviewing techniques is both feasible and effective in significantly decreasing depression and enhancing resilience in this sample of low-income, second-generation, U.S. born Latinas
Bernal et al. [23]	Can cognitive behavioral therapy be optimized with parent psychoeducation? A randomized effectiveness trial of adolescents with major depression in PR	RCT	2019	RCT	Clinic in a metropolitan area in CA	RCT	121	Depression severity ratings over time	CDI, CDRS-R, SIQ-Jr	CBT+TEPSI, a parental psychoeducational intervention, was not found to optimize depression scores compared to CBT alone
Kataoka et al. [24]	A school-based mental health program for traumatized Latino immigrant children	Interventional	2003	Quasi-experimental study	Emigrated Spanish-speaking students recruited from nine schools with the Emergency Immigrant Education Program	Clinical trial	198	PTSD and depressive symptoms	CPSS, child depressive index	Significant decrease in CDI and CPSS scores of participants in the trauma-focused CBT intervention arm. No significant decrease in the control group scores
Mufson et al. [25]	A pilot study of brief IPT-A delivered in primary care	Pilot open clinical trial	2015	Controlled clinical trial	Urban pediatric primary care clinics in NY	Controlled clinical trial	10	Depression severity ratings	BDI-II, CDRS-R, Clinical Global Impression scale, Global Assessment Scale for Children, Social Adjustment Scale - Self-Report	Significant improvement was found across all measure outcomes for patients who participated in IPT-A
Schmaling and Hernandez [26]	Problem-solving treatment for depression among Mexican Americans in primary care	Feasibility study	2008	Interventional clinical trial/feasibility	Mexican-American patients recruited from two rural primary care clinics in Fabens and Montana Vista, TX, with diagnoses of depression	Interventional clinical trial/feasibility	146	Depression severity ratings	HSCL-20	Patients who participated in PST-PC had depressive symptoms score decrease over the course of treatment, with a minority of participants who participated for four or more sessions seeing the greatest decrease
Kanter et al. [27]	Initial outcomes of a culturally adapted behavioral activation for Latinas diagnosed with depression at a community clinic	Controlled clinical trial, but control arm outcomes were not measured.	2010	Open trial	Sixteenth Street Community Health Center in Milwaukee, WI	Open trial	24	Depression severity ratings	BDI-II, HRSD	Study found support for the feasibility and effectiveness of BAL therapy for depressed Latinos
Kanter et al. [28]	A randomized hybrid efficacy and effectiveness trial of behavioral activation for Latinos with depression	RCT	2015	RCT	Sixteenth Street Community Health Center in Milwaukee, WI	RCT	43	Depression severity ratings	25-item modified HRSD, BDI-II	BAL performed well with regard to treatment engagement and retention, and there was an interaction between number of sessions and condition, with those who were engaged with and participated in more sessions having significant reductions in depression
Pérez-Rojas et al. [29]	Psychotherapy outcomes with Latinx clients attending Hispanic-serving institutions and predominantly White institutions	Retrospective analysis	2023	2013-2015 Center for Collegiate Mental Health dataset	Latinx students seeking treatment in college/university counseling centers	Retrospective cohort study	9515	Depression, academic distress, and generalized anxiety	Depression, generalized anxiety, and academic distress subscales of CCAPS-34	Latinx students seeking therapy in HSIs versus those in PWI's had greater relief of symptoms of academic distress but not depressive symptoms or generalized anxiety
Collado et al. [30]	Pilot of the brief behavioral activation treatment for depression in Latinos with limited English proficiency: preliminary evaluation of efficacy and acceptability	Controlled clinical trial	2014	Controlled clinical trial	Unspecified	Controlled clinical trial	10	Depression severity ratings	BDI-II, BADS	Study found that Latinos with limited English proficiency showed decreases in depressive symptomatology and were willing to participate in BATD
Collado et al. [31]	The efficacy of behavioral activation treatment among depressed Spanish-speaking Latinos	RCT	2016	RCT	Unspecified	RCT	46	Depression severity ratings	BDI-II, BADS, TAC	BATD resulted in greater decreases in depressive symptoms over time and a greater major depressive disorder remission than supportive counseling alone, as well as greater increases in activity level and environmental reward for an underserved U.S. ethnic minority population
Comas-Díaz [32]	Effects of cognitive and behavioral group treatment on the depressive symptomatology of Puerto Rican women	RCT	1981	RCT	Unspecified	RCT	12	Depression severity ratings	BDI, HRSD, DBRS	Both therapy groups improved significantly over the course of treatment, but behavioral group therapy subjects maintained improvement at follow-up compared to cognitive group therapy
Alegría et al. [33]	Effectiveness of the ECLA intervention in low-income Latinos	RCT	2014	RCT	Face-to-face/telephone delivery at eight community health clinics in Boston, MA, and San Juan, PR	RCT	257	Depression severity	PHQ-9, HSCL-20	Reduced depression severity in patients who received telephone or face-to-face ECLA. There is higher treatment initiation for telephone ECLA versus face-to-face ECLA
Alcántara et al. [34]	Treatment moderators and effectiveness of engagement and counseling for Latinos intervention on worry reduction in a low-income primary care sample	Retrospective analysis	2016	RCT	Telephone calls and primary care clinics in Boston, MA and San Juan, PR	Secondary analysis	257	Worry symptom ratings	PSWQ	Patients receiving ECLA had a greater reduction in worry symptoms when compared to usual care group members. Furthermore, the telephone version of ECLA produced greater decreases compared to the face-to-face condition
Dwight-Johnson et al. [35]	Telephone-based cognitive behavioral therapy for Latino patients living in rural areas: a randomized pilot study	RCT	2011	RCT	Telephone calls to patients in Yakima Valley Farm Workers Clinic	RCT	101	Depression severity ratings	Hopkins SCL, PHQ-9	Patients who received CBT over the telephone were more likely to experience improvement in depression scores over a six-month follow-up period compared to patients who received enhanced usual care
Moreno et al. [36]	Use of standard Webcam and Internet equipment for telepsychiatry treatment of depression among underserved Hispanics	Improvement in depressive symptoms; RCT	2012	RCT	Video appointments and a community health center	RCT	167	Depressive severity ratings	MADRS, Q-LES-Q, PHQ-9, SDS	Use of measurement- and algorithm-based treatment for depression by psychiatrists via ebcam can lead to improvements in depression severity, functional ability, and quality of life that are at least equivalent and possibly superior to improvements achieved with treatment as usual delivered by primary care providers
Kafali et al. [37]	Cost-effectiveness of a randomized trial to treat depression among Latinos	Cost-effectiveness	2014	RCT	Telephone calls and clinic visits in both Boston, MA, and San Juan, PR	RCT	257	Cost measurement and depression severity ratings	PHQ-9, HSCL-20	Both CBT over the phone and face-to-face were associated with significantly reduced scores on the PHQ-9 and HSCL-20, compared with usual care. However, CBT over the phone was significantly less costly and more cost-effective compared to CBT offered face-to-face
Dahne et al. [38]	Pilot randomized controlled trial of a Spanish-language behavioral activation mobile app (¡Aptívate!; College Park, MD: Behavioral Activation Tech, LLC) for the treatment of depressive symptoms among United States Latinx adults with limited English proficiency	RCT	2019	RCT	Mobile apps	RCT	42	App feasibility, depression severity ratings	PHQ-8, BDI-II	Study found significant interaction between app usage and treatment, with ¡Aptivate! users reporting lowered depressive symptomatology over time compared with the treatment-as-usual group

Ell et al. analyzed the sustainability of the effects from their 2010 randomized controlled trial (RCT) at two years [20]. A total of 264 patients from the previous 2010 RCT participated. At 24 months follow-up, more INT than EUC participants received ongoing antidepressant treatment (38% versus 25%, chi-square=5.11, df=1, p=0.02); INT patients also experienced improved SCL-20 scores (adjusted mean difference: -0.22, p=0.001) (SCL-20: <0.5 {adjusted OR=2.06, 95% CI=1.09-3.90}, p=0.03) and Patient Health Questionnaire (PHQ) scores. From these findings, researchers concluded that socioculturally tailored collaborative care (maintenance antidepressant medication, symptom monitoring, behavioral activation relapse prevention) is effective in managing depression in Latinos with diabetes in the safety net setting. This 24-month follow-up analysis offered good evidence for this claim, although interpretation is limited by potential attrition bias.

Hay et al. analyzed quality of life and depression free days outcomes from the Ell et al. trial [20,21]. Multifaceted Diabetes and Depression Program (MDDP) participants experienced statistically significant improvements in quality-adjusted life years (QALYs), utility (per SF-12 utility scale), and depression free days (per PHQ-9). In terms of QALYs, the MDDP group exhibited an average 0.13-QALY increase compared with the control group over 18 months, giving an incremental cost per QALY of $4053. MDDP patients also enjoyed 43 more depression-free days than propensity-score matched controls on multivariable logistic regression analysis (p<0.001). From these results, the authors concluded that socioculturally and organizationally adapted collaborative care is cost-effective in improving quality of life in a low-income, predominantly Hispanic population.

Heileman et al. shared results from a controlled clinical trial also examining the efficacy of a psychotherapy intervention in treating depression among eight low-income, second-generation Latinas (n=8), noting that women and US-born Latinx people have relatively higher rates of depression [22]. The intervention consisted of schema therapy, a form of therapy focused on connecting childhood sources of psychological challenges to current issues, combined with motivational interviewing techniques and collaborative mapping, with patients physically drawing out mental dynamics. The authors applied motivational interviewing, a counseling style that stresses guiding patients to internal reasoning, towards a personal sense of urgency and confidence about completing treatment. These techniques resulted in significantly decreased Beck Depression Inventory (BDI-II) scores and significantly increased resilience scores among the study population over the eight-week treatment program compared with baseline. In particular, there was a mean difference of 28.3 (SE=4.0) in BDI-II scores from session one to session eight and a mean difference of 7.8 (2.3) from session eight to 12-month follow-up. Regarding resilience scores, there was a mean difference of -31.5 (8.8) from session one to session eight and -9.5 (4.3) from session eight to the 12-month follow-up. There were no significant differences in BDI-II scores at the three- and 12-month follow-up visits. The researchers concluded that this combined depression care model is effective in treating depressive symptoms and enhancing resilience in low-income, second-generation Latinas in the U.S. As a controlled clinical trial with a very small sample size (n=8), this study provides exploratory but encouraging evidence for this claim.

Bernal et al. investigated whether the efficacy of cognitive behavioral therapy (CBT), a style of therapy focused on adjusting thinking and behavioral patterns, for depressive symptoms could be improved by including a parent psychoeducation intervention, Talleres de Educacion Psicologica (TEPSI) [23]. TEPSI is a group intervention comprising eight educational sessions to inform parents about adolescent depression. Their randomized controlled trial included 121 Puerto Rican adolescents and utilized a variety of instruments to measure outcomes, including the Children’s Depression Inventory (CDI). Participants were randomized to individual CBT with or without parent psychoeducation. The researchers did not find significant improvement in participants’ depressive symptoms when CBT was paired with TEPSI (CDI score change p=0.376; CDRS-R p=0.621; Suicidal Ideation Questionnaire-Junior {SIQ-Jr} p=0.399). However, the researchers found a main effect of culturally adapted CBT over time - 76.7% of adolescents in the CBT-only group and 80.4% in the CBT+TEPSI group achieved an absolute reliable change score (RCI) above 1.96. Bernal et al. concluded that parent psychoeducation did not accelerate the rate of depressive symptom reduction in the participants, and both intervention arms arrived at roughly the same clinical endpoint [23]. As a well-designed randomized controlled trial, this study provides relatively high-level evidence regarding the effectiveness of culturally adapted CBT both with and without parent psychoeducation for depressive symptoms in Latinx adolescents. To further inform our understanding of the role of parental involvement in CBT, future researchers may consider studying a higher level of parental involvement than simple psychoeducation.

Also investigating the efficacy of behavioral therapy in children, Kataoka et al. reported the results of a quasi-experimental study with randomized features in 198 Latino immigrant children who had trauma-related depression and/or post-traumatic stress disorder (PTSD) [24]. Participants were randomized to an intervention group or a waitlist control. The intervention consisted of cognitive behavioral therapy (CBT) with a focus on trauma, delivered in Spanish by bilingual, bicultural school social workers over the course of eight sessions. Intervention participants experienced a significant decrease in mean Children’s Depression Inventory (CDI) score at three-month follow-up, decreasing from a mean CDI score of 16 to 14. Additionally, the intervention group experienced a significant decrease in the mean Child PTSD Symptom Scale (CPSS) score at follow-up, from a mean score of 19 to 13. The waitlist group experienced non-significant decreases in mean CDI and CPSS scores. The researchers concluded that school-based, trauma-focused CBT is associated with a modest decrease in PTSD and depressive symptoms in Latino immigrant students. In the future, the researchers may consider testing the efficacy of this intervention in second- or later generation-immigrant students. As a quasi-experimental study with a waitlist comparison group, this study provides moderate-level evidence for the conclusion made, with strengths including a relatively large sample and comparison group.

Mufson et al. examined the preliminary efficacy of a brief version of interpersonal psychotherapy for depressed adolescents (BIPT-A) in 10 low-income Latino adolescents [25]. All participants received six weekly sessions of BIPT-A. BIPT-A is a brief version of interpersonal therapy for adolescents, consisting of psychoeducation about depression and communication/problem-solving skills. According to the study, interpersonal therapy for adolescents is "based on the premise that depression commonly occurs within an interpersonal context and aims to reduce depression by helping adolescents improve their relationships and social interactions. Depression outcomes were measured via BDI-II and the Children’s Depression Rating Scale-Revised (CDRS-R). Participants attended a mean of 4.9 sessions (SD=1.29), and all rated the treatment as “very helpful.” Participants' depressive symptoms significantly improved with treatment (post-treatment BDI-II t=3.79, p=0.004; CDRS-R t=3.02, p=0.015). The researchers concluded that a brief version of IPT-A was an acceptable and effective treatment for Latino adolescents with depression. This study provided exploratory but promising evidence for these claims.

Schmaling and Hernandez analyzed the results of a single-group effectiveness study of behavioral therapy for depression in 146 Mexican American patients with depressive symptoms in rural Texas [26]. A total of 117 participants agreed to treatment and received eight sessions of problem-solving treatment for primary care (PST-PC). PST-PC is a brief, skills-based behavior therapy to treat depressive symptoms in the primary care setting; it approaches depression as a result of problems encountered in daily life, helping patients identify and address these problems. Depression symptoms were measured via the 20-item Hopkins Symptom Checklist (HSCL-20). All research staff and therapists were Latino. Participants who attended four or more PST-PC sessions (n=55) experienced significantly larger decreases in HSCL scores at three-month follow-up than those who completed three or fewer sessions. Specifically, participants who attended four or more sessions declined by an average of 0.86±0.97 points on the scale, whereas those who attended fewer than four sessions declined by 0.40±0.66 points. The researchers concluded that although a significant decrease in symptoms was observed, mental health practitioners must also acknowledge the Latino cultural values and traditions that may impact therapy delivery. This study’s main strength was its large sample size to lend evidence to the efficacy of using PST-PC for Latinx patients with depressive symptoms. In future studies, the research group may consider exploring how incorporating different cultural values, such as familism or simpatía, may affect intervention efficacy.

Kanter et al. conducted a pilot evaluation of culturally/linguistically adapted behavioral activation for Latinos (BAL) [27]. Behavioral activation is a form of therapy focused on using positive reinforcement to help patients engage in meaningful activities to treat depressive symptoms. Participants were Latino patients (n=24) recruited from an urban bilingual community health center and were assigned to the BAL arm or control arm (not used for comparison). A total of 10 Latinas received 12 sessions of BAL therapy, and depression outcomes were measured via BDI-II and Hamilton Rating Scale for Depression (HRSD). Therapists reported that most BAL homework assignments were partially completed at the next session (84% of sessions), and a median of 10 sessions were completed over a median of 16 weeks. Additionally, BDI-II and HRSD scores significantly decreased post-treatment (BDI-II t{5}=5.69, p=0.02, d=1.67/HRSD t{5}=12.77, p<0.001, d=1.57). In conclusion, researchers found that culturally tailored BAL is an effective and feasible treatment for Latinos with depression. Subsequent research should analyze outcome differences between culturally adapted BAL and non-culturally adapted BAL and conduct a sub-analysis of the most effective traits of the culturally adapted BAL. This trial was well-designed but limited in scope with a small size as a pilot study; the strong observed effect size was a particularly promising reason to further explore BAL in Latinx patients.

Kanter et al. built upon this previous study, conducting a randomized controlled trial investigating the efficacy of a depression intervention in low-income Latinos [28]. Of 43 eligible participants, 21 were randomized to the behavioral activation (BAL) arm and received 12 sessions of behavioral activation therapy. The rest received treatment as usual (TAU) for 12 sessions. Depression severity was measured using the Beck Depression Inventory-II (BDI-II) and the first 17 items of the Spanish version of the HRSD. The BAL group attended a mean (M) of 8.21 sessions (SD=3.95) and was more likely to complete therapy than the TAU group (M=4.95, SD=3.41). BAL participants who attended nine to 12 sessions experienced a significant decrease in HRSD scores, t(8)=5.97, p<0.001, d=1.99. However, for patients who only attended 0-4 sessions, the effect size favored TAU. The researchers concluded that behavioral activation therapies could be utilized to improve treatment retention and outcome in Latinos compared with treatment as usual. As a randomized controlled trial with a more robust sample size, this follow-up offered strong evidence for this appropriately circumscribed conclusion.

Similarly, Pérez-Rojas et al. compared the efficacy of psychotherapy provided by university/college counseling centers (UCCs) on Latinx students in Hispanic-serving institutions (HSIs) and in Predominantly White institutions (PWIs) [29]. The study involved researchers examining a large 2013-2015 national data set (CCMH) of Latinx students who received <13 psychotherapy sessions at UCCs. Depression and anxiety outcomes were measured by the Counseling Center Assessment of Psychological Symptoms (CCAPS-34). Researchers utilized multilevel modeling to predict depression, generalized anxiety, and academic distress over time. Depression and generalized anxiety outcomes did not vary by HSI status. However, participants attending HSIs experienced significant improvement in academic distress compared with participants at PWIs (β = -0.11, p=0.003 in multivariable multilevel modeling). Researchers hypothesized that the shared culture, community, and academic representation at HSIs contributed to students’ improvements in academic distress. With a retrospective secondary analysis design, this study presented limited evidence of increased psychotherapy efficacy at HSIs compared to PWIs.

Collado et al. analyzed the preliminary results of a controlled clinical trial of brief behavioral activation treatment for depression in 10 Latinos with limited English proficiency and depressive symptoms [30]. The participants received 10 sessions of therapy based on literal Spanish translations of Brief Behavioral Activation Treatment for Depression (BATD). BATD is a brief version of behavior activation therapy and treats depressive symptoms by encouraging patients to participate in meaningful and rewarding activities. The trial found a significant decrease over time in depressive symptomatology, as measured by the BDI-II (β = -1.64, SE=0.21, p<0.001), and an increase in activation levels, as measured by the Behavioral Activation for Depression Scale, activation subscale (BADS) (β=0.86, SE=0.35, p=0.04). The research group concluded that BATD improves depressive symptomatology in this group. For a small (n=10) and controlled clinical trial, this study presented limited but promising evidence for the use of BATD in Latinx patients with depression.

Given these results, a randomized clinical trial of the Collado et al. study was conducted in 2016 [31]. This study consisted of 46 Spanish-speaking Latino participants with depression. Participants were randomized to BATD or supportive counseling (SC), which emphasizes listening without providing advice or skills. The participants in the BATD condition experienced a significant reduction in depressive symptoms, greater rates of remission of depression, and greater increases in activity level over time than those in the SC condition. Among participants who completed treatment, those in the BATD group (93.3% of n=15) showed greater depression remission rates (𝜒^2^{1}=6.52, p=0.01) than those in SC (50% of n=12) and a significant decrease over time on depressive symptomatology (β = -1.64, SE=0.21, p<0.001). From this trial, researchers concluded that BATD is an effective treatment to decrease depression in this group. In the future, researchers may consider increasing the follow-up period for this study, as participants were followed up only a month after treatment. While limited in duration, this randomized clinical trial offered good evidence for the use of BATD over SC for Latino patients with depression.

Prior to these studies, Comas-Díaz conducted a randomized controlled trial to compare behavioral therapy, cognitive therapy, and no treatment (waiting list group) in low-income Puerto Rican women with depression (n=12) [32]. The therapist was a bilingual, Puerto Rican woman. In this trial, behavioral therapy worked to reduce depression by helping patients engage in rewarding activities, while cognitive therapy focused on identifying and modifying negative thoughts that contributed to depression. Clients in the therapy groups experienced significantly less depression than the waiting list group according to reductions in the Beck, Hamilton, and Depressive Behavior Rating Scales (DBRS). Specifically, clients in both therapy groups reported fewer depression symptoms on the Beck scale, F(1, 13)=10.302, p<0.01, and on the Hamilton scale, F(1, 13)=14.854, p<0.01. However, no significant differences in depressive symptoms between the no-treatment and therapy groups were found. There was also no significant difference between the behavioral and cognitive therapy groups. Although this study used a randomized controlled trial design, its age range and sample size limit its generalizability.

Telepsychiatric therapy interventions: Telepsychiatric interventions offer a promising new manner to improve mental health in Latinx patients. Alegría et al. conducted an RCT to evaluate the efficacy of the Engagement and Counseling for Latinos (ECLA) intervention in low-income Latinos [33]. This intervention consisted of six to eight culturally tailored CBT/care management sessions delivered via face-to-face or telephone (ECLA-F, ECLA-T). ECLA combined CBT and care management, encouraging patients to participate in rewarding activities, challenge negative thought patterns, and develop coping strategies. The participants were 257 Latino patients recruited from eight community health clinics in Boston, MA, and San Juan, PR. Usual care consisted of the clinic standard of care (watchful waiting, antidepressant/anxiolytic prescription, or mental health referral). ECLA-T and ECLA-F PHQ-9 and HSCL scores improved significantly compared to usual care (PHQ-9 ECLA-F p<0.05; effect size {ES}=0.56 and ECLA-T p=0.01; ES=0.64/HSCL ECLA-F p<0.05; ES=0.32 and ECLA-T p<0.05; ES=0.34). There was no significant difference in PHQ-9 and HSCL scores between ECLA-F and ECLA-T (p=0.89 and 0.69). Additionally, researchers found that ECLA-T resulted in higher treatment initiation. This large-sample, well-designed randomized controlled trial provided strong evidence supporting the use of ECLA-F and ECLA-T for Latinx patients.

A secondary analysis by Alcántara et al. evaluated the preceding trial data to determine if the Engagement and Counseling for Latinos (ECLA) intervention reduced worry symptoms co-occurring with depression [34]. The group also attempted to determine whether treatment delivery method and sociodemographic characteristics impacted treatment efficacy. Treatment efficacy was evaluated via PHQ-9 scores and Penn State Worry Questionnaire (PSWQ). Participants in telephone and face-to-face ECLA conditions experienced reductions in worry at four months relative to those in usual care (PSWQΔ = -3.28, p<0.05). However, patients in the telephone ECLA condition (M = -7.83, SD=11.45) experienced greater worry reduction than those in the face-to-face condition (M = -6.73, SD=12.23; p<0.05). Meanwhile, participants in the face-to-face ECLA condition did not experience a significant difference in worry symptoms compared to usual care. From these findings, the researchers concluded that ECLA is effective in decreasing worry symptoms in a low-income Latino population and that treatment via telephone can be an even more effective delivery method than face-to-face. This secondary analysis of the previous randomized controlled trial added to the weight of evidence supporting the use of ECLA with Latinx patients, whether in-person or via telehealth.

Dwight-Johnson et al. conducted a randomized controlled trial to evaluate the efficacy of culturally tailored, telephone-based cognitive behavioral therapy (CBT) in a rural, Latino population [35]. The group compared the effects of enhanced usual care versus eight sessions of telephone-based CBT for improving depression outcomes in 101 Latino patients, using the Hopkins Symptom Checklist (SCL) depression items and the Patient Health Questionnaire-9 (PHQ-9). Enhanced usual care consisted of provider usual care for depression, including antidepressants or referral to outside services. The CBT provided in the intervention group was culturally tailored, stressed behavioral activation, and attempted to mitigate negative thoughts. The CBT group experienced greater reductions in their PHQ-9 and SCL scores compared with enhanced usual care over a six-month follow-up period: β = -0.41, t = -2.36, df=219, p=0.018, for the SCL; and β = -3.51, t = -2.49, df=221, p=0.013 for the PHQ-9. These patients were also more likely to report satisfaction with depression treatment than those in the enhanced usual care arm (p=0.013). From these results, the researchers concluded that CBT is helpful in increasing access to mental health services in a rural Latino population. This well-designed randomized controlled trial provided convincing evidence as a pilot study for the use of CBT tailored towards rural Latino populations.

Another study investigating the efficacy of telehealth to increase Latinos’ access to mental health services was conducted by Moreno et al. [36]. In this study, the researchers compared the efficacy of depression treatment provided by a psychiatrist remotely via webcam versus usual depression treatment by a primary care provider in 167 Hispanic patients. Improvement in depression was measured by ratings on the Montgomery-Åsberg Depression Rating Scale (MADRS), self-ratings on the PHQ-9, the Quality of Life Enjoyment and Satisfaction Questionnaire (Q-LES-Q), and the Sheehan Disability Scale (SDS). Patients in the webcam intervention had six monthly remote appointments with a bilingual psychiatrist. Intervention treatment included medication management, psychoeducation, and other brief supportive interventions as needed. Usual treatment consisted of antidepressants and counseling by behavioral health clinicians on site. The webcam intervention further reduced depression in patients, as compared to the treatment-as-usual group (MADRS F=5.93, df=2 and 244, p<0.01; PHQ-9 F=3.6, df=2 and 260, p<0.05). Similarly, both groups experienced improved quality of life and functional ability scores (Q-LES-Q F=112.16, df=2 and 130, p<0.01; SDS F=129.43, df=2 and 131, p<0.01), however both of these measures also demonstrated a significant main effect of time, favoring the webcam intervention (Q-LES-Q F=3.1, df=2 and 260, p<0.05; SDS F=3.23, df=2 and 262, p<0.05). Researchers concluded that a webcam-based psychiatry intervention can improve depression, quality of life, and functional ability in a Latino patient population. While this study employed random assignment with a large sample size, its design lacks a true control group, with acknowledged limitations of the significant differences for interventions between samples (namely, patients in the webcam group were seen more often and their visit was dedicated to depression treatment, whereas patients in the treatment-as-usual group were seen at primary care visits).

A couple of years later, Kafali et al. published results from an RCT comparing the cost-effectiveness of conducting CBT over the phone or face-to-face versus usual primary care for Latino patients, as well as efficacy data [37]. The study consisted of 257 Latino patients randomized to the following three arms: usual care, telephone CBT, or face-to-face CBT. Usual care consisted of primary care. Both CBT groups received their usual primary care. The CBT consisted of six to eight sessions, including psychoeducation on depression, activities to decrease depressed mood, and challenging negative thoughts. Cost-effectiveness of these interventions was measured as incremental cost-effectiveness ratios (ICERs). Mental health outcomes were measured by the PHQ-9 and Hopkins Symptom Checklist (HSCL). In terms of cost, the researchers found that both CBT interventions were significantly more costly than usual care. However, both intervention groups were associated with significant improvement in depression compared with usual care (face-to-face CBT PHQ-9: -2.30, p=0.020, HSCL: -0.27, p=0.031; phone CBT PHQ-9: -2.98, p=0.003, HSCL: -0.33, p=0.010). Compared to face-to-face CBT, phone CBT was significantly less costly (by $501). In conclusion, researchers found that phone CBT was effective in treating depression in Latino patients and was more cost-effective than traditional face-to-face therapy. This robust randomized clinical trial presented strong support for the cost-effectiveness of phone-based CBT.

Later, researchers began to investigate the use of phone applications (apps) to deliver mental healthcare to Latino patients. For context, therapist-delivered telepsychotherapy involves real-time treatment provided remotely by a clinician over the internet, whereas mental-health interventions delivered through an app or online program are self-guided and completed independently without clinician or therapist involvement. Dahne et al. conducted an RCT to evaluate the feasibility and efficacy of a Spanish-language behavioral activation mobile app (¡Aptívate! {College Park, MD: Behavioral Activation Tech, LLC}) in treating depressive symptoms in Latino patients with limited English proficiency [38]. In the study, 42 Latino patients were randomized to either ¡Aptívate!, an active control CBT Spanish-language app (iCouch CBT), or treatment-as-usual (no mobile app, but participants could continue their usual mental healthcare). The app functioned as a self-help intervention; participants independently completed behavioral activation activities, such as scheduling rewarding activities for themselves, in the app. Feasibility was measured via app utilization and app analytics data, and depressive symptoms were measured via PHQ-8 and BDI-II scores. Researchers found that self-reported app utilization was significantly higher for ¡Aptívate! as compared to iCouch CBT, and in general, app retention was high for ¡Aptívate!. Additionally, ¡Aptívate! participants experienced an average 13.79 (SD: 12.33) decrease in BDI-II score post-treatment, and iCouch participants had an 11.25 (8.19) point decrease. Meanwhile, participants in the TAU condition had an 8.0 (5.44) point decrease. From this study, researchers found that a behavioral activation, self-help mobile app (¡Aptívate!) was feasible and effective in reducing depressive symptoms in a Latino population.

Part 2: Therapeutic Interventions to Treat Substance Use Disorders in Hispanic/Latinx U.S. Populations 

Conventional therapy interventions: Several psychotherapy trials have been tested to treat substance use disorders in Latinx populations (Table 2). Carrol et al. conducted a randomized controlled trial to compare the efficacy of a previously tested motivational enhancement therapy (MET) to counseling as usual (CAU) for Hispanic substance users [39]. In this trial, MET consisted of three sessions of motivational interviewing techniques aimed at exploring patients’ perceptions of their substance use and strengthening their motivation to change their behavior. Participants were recruited from five outpatient substance abuse treatment programs that were offered in Spanish within the Clinical Trials Network. Researchers were interested in comparing treatment retention and frequency of substance use for patients in both groups, which was measured through both self-reported drug usage and urinalysis. A total of 66% of patients completed all three protocol sessions and both interventions, resulting in reductions in substance use across both measures during the four-week therapy phase. No significant relationship emerged between treatment condition by time or site by treatment condition. However, they did replicate a finding from another study with MET that found it had a moderate, significant effect for those whose problem substance was alcohol. This randomized controlled trial effectively supported the authors’ conclusions that both individual treatments in Spanish were effective in treating substance users, but the differential effectiveness of MET may be limited to those whose primary substance is alcohol.

**Table 2:** Psychotherapy trials for substance use disorders in Hispanic/Latinx U.S. populations. CAU: counseling as usual; MET: motivational enhancement therapy; SCID: Structured Clinical Interview for DSM Disorders; TLFB: timeline follow-back; ARSMA-II: Acculturation Rating Scale for Mexican Americans-II; MEIM: Multigroup Ethnic Identity Measure; FS: Familism Scale; CT: Connecticut; CBT: cognitive behavioral therapy; CSQ: Cognitive Style Questionnaire; S-CBT: Standard Cognitive Behavioral Treatment; A-CBT: Accommodated Cognitive Behavioral Treatment; CBT4CBT: computer-based training for cognitive behavioral therapy; CIFFTA: culturally informed and flexible family-based treatment for adolescents; TFT: traditional family therapy; LGBT: Lesbian, Gay, Bisexual, and Transgender; LA: Los Angeles; FL: Florida; RBPC: Revised Behavior Problem Checklist; ASI: Addiction Severity Index; FES: Family Environment Scale; SFSR: Structural Family Systems Rating; BSFT: brief strategic family therapy; GC: group treatment control

Studies	Study title	Study type	Publication year	Data source	Setting	Study design	Study, n	Main outcome	Measure instrument	Main findings
Carrol et al. [39]	A multisite randomized effectiveness trial of motivational enhancement therapy for Spanish-speaking substance users	RCT	2009	RCT	Outpatient substance abuse treatment programs	RCT	405	Primary retention and days of substance use per week	Self-reported drug usage, urinalysis	Both interventions, CAU and MET, saw reductions in substance use; however, there was no significant relationship between treatment condition by time or site by treatment condition
Burge et al. [40]	An evaluation of two primary care interventions for alcohol abuse among Mexican-American patients	RCT	1997	RCT	Outpatient facility	RCT	175	Severity scores of alcohol abuse	Addiction Severity Index, Blood tests	All groups, both control and treatment, demonstrated significant improvement, without a significant difference in effectiveness between treatment and control conditions
Burrow-Sanchez and Wrona [41]	Comparing culturally accommodated versus standard group CBT for Latino adolescents with substance use disorders: a pilot study	Randomized controlled trial	2012	RCT	Community center	RCT	35	Feasibility and adolescent substance use	SCID, TLFB, CSQ, ARSMA-II, MEIM, FS	S-CBT and A-CBT demonstrated similar satisfaction and retention rates, with both showing a significant decrease in substance use. It was also found that ethnicity and familism were moderating substance use outcomes
Burrow-Sánchez and Hops [42]	A randomized trial of culturally accommodated versus standard group treatment for Latina/o adolescents with substance use disorders: post-treatment through 12-month outcomes	RCT	2019	RCT	Community center	RCT	70	Adolescent substance use	SCID, TLFB ARSMA-II, MEIM, FS	There was a significant difference in adolescent substance use, with the culturally accommodated treatment showing greater decreases. It was also found that familism moderated treatment outcome
Santisteban et al. [43]	Efficacy of brief strategic family therapy in modifying Hispanic adolescent behavior problems and substance use	RCT	2003	RCT	In-office setting in Miami, FL	RCT comparing the efficacy of brief strategic family therapy versus group treatment in modifying Hispanic adolescent behavior problems and substance use	126	Adolescent conduct problems and delinquency, adolescent-reported drug use, observer and self-reports of family functioning, and peer-based delinquency	RBPC, ASI, FES, and SFSR, and urine toxicology reports	BSFT was significantly more efficacious than the GC at improving conduct problems, peer-based delinquency, and self-reported drug use. BSFT was significantly more efficacious than the GC in improving family functioning
Santisteban et al. [44]	Preliminary results for an adaptive family treatment for drug abuse in Hispanic youth	RCT	2011	RCT	Participants were referred by a local Juvenile Addictions Receiving Facility and by Department of Juvenile Justice Diversion programs in Miami, FL. Intervention setting not found	RCT comparing efficacy of the CIFFTA and traditional family therapy	28	Adolescent-reported drug use (marijuana+cocaine), parent-reported behavior problems, adolescent-reported behavior problems, and adolescent-reported and parent-reported parenting practices	Revised Behavior Problems Checklist, the Youth Self-Report; and drug use was based on the timeline follow-back over a 31-day period and verified via urine toxicology	CIFFTA led to significant reduction in self-reported drug use and significant improvement in adolescent perceptions of parenting practices
Tucker et al. [45]	Pilot evaluation of a brief intervention to improve nicotine patch adherence among smokers living with HIV/AIDS	RCT	2017	RCT	One of multiple locations of a social service agency serving the LGBT community in LA	RCT comparing efficacy of a standard 5 A's counseling session and eight-week treatment of nicotine patch versus a similar intervention that includes a 10-min module focused on improving adherence to the nicotine patch	40	7-day point prevalence and 90-day continuous abstinence from cigarette smoking; patch usage	Self-report, verified by expired air carbon monoxide. Patch counting at follow-up	Results are promising, suggesting that an added 10-min module on nicotine patch adherence to a standard 5 A's protocol can increase abstinence rates
Paris et al. [46]	Culturally adapted, Web-based cognitive behavioral therapy for Spanish-speaking individuals with substance use disorders: a randomized clinical trial	RCT	2018	RCT	3 outpatient clinics in New Haven, CT	RCT comparing efficacy of a culturally adapted version of CBT4CBT to standard outpatient mental health and addiction treatment in substance abuse treatment-seeking Latino adults	92	Frequency of primary substance use over time	Self-report with biologic verification (urine analysis and breathalyzer)	Patients in the CBT4CBT arm had a significantly greater reduction in days of their primary substance use over time than those who received standard treatment only

Decades prior, Burge et al. conducted a randomized controlled trial to examine two alcohol abuse primary care interventions’ effects (a physician intervention and a clinic-based six-week psychoeducational group) on drinking patterns and psychosocial problems assessed by the Addiction Severity Index (ASI) [40]. The participants were recruited from a family outpatient clinic and included 175 Mexican American female and male patients diagnosed as having alcohol abuse, dependence, or neither based on DSM-III guidance. Participants were separated into four groups, which meant that they either received one treatment, both treatments, or no treatment; all were given the choice of receiving a treatment in English or Spanish. Researchers found that participants in all four treatment groups showed significant improvement over time across the outcomes of interest, with little difference in improvement between interventions and control groups. Researchers concluded that the two interventions offered no significant advantage. This study employed a randomized controlled trial design with a robust sample size, presenting sufficient evidence that neither treatment had superior clinical efficacy.

Burrow-Sánchez and Wrona conducted a randomized controlled trial, where 35 Latino adolescents, 94% male, with substance abuse or dependence as assessed by the Structured Clinical Interview for DSM-IV, were randomized to either an existing standard version of cognitive behavioral substance abuse treatment (S-CBT) or to a culturally accommodated version (A-CBT) for 12 weeks [41]. S-CBT was a standard 12-session group CBT intervention designed to help patients improve coping skills. A-CBT was also a group CBT intervention, but it incorporated Latino cultural values and concerns, such as familism. The study sought to determine the feasibility and relative efficacy of both interventions and looked at retention and satisfaction rates via the Client Satisfaction Questionnaire (CSQ) among the participants in each group, as well as self-reported substance use rates over time. They found that the interventions had no significant difference in retention (78% {14/18} for S-CBT and 82% {14/17} for A-CBT) and adolescent satisfaction rates of 61.27 (6.36) out of a max of 72 mean for S-CBT and 55.92 (9.56) mean for A-CBT. Using linear mixed model (LMM) analysis, researchers found that participants in both groups showed significant decreases in substance use pre- to post-treatment in their statistical modeling, where the dependent variable for all models was the percentage of days out of 90 where drug usage excluding tobacco was reported. This study possessed a small sample size (n=35) with a substantial skew towards male participants and lacked a control group, offering fair evidence for the utility of either of S-CBT or A-CBT in Latinx populations.

Six years later, Burrow-Sánchez and Hops reported another randomized controlled trial to compare the efficacy of S-CBT to A-CBT [42]. The participants for this study were 70 Latina/o (90% male) adolescents with diagnosed drug abuse or drug dependence according to DSM-IV-TR. Researchers used substance use as the main measure of efficacy, measured as the number of days substances were used in the past 90 days before an assessment. Using a Poisson model, they found that there was a significant between-group difference (t{273.5}=3.86, p=0.0001, d=0.92, 95% CI: 0.43, 1.42) at the 12-month time point in substance use, which showed lower mean substance use days in A-CBT than in S-CBT. Treatment outcome was moderated by scores of parental familism, with children of parents who scored low or high on familism in the treatment condition reporting significantly greater decreases in substance use than children of parents with similar scores in the standard condition. Researchers concluded that the culturally accommodated treatment differentially improved outcomes compared to the standard treatment for Latina/o adolescents with substance use disorders, and that parental familism is an important moderator for outcomes. This follow-up improved upon the previous study design by collecting a larger sample and presented some evidence for the utility of A-CBT over S-CBT for Latinx adolescents.

Santisteban et al. investigated the efficacy of brief strategic family therapy (BSFT) in modifying behavior problems and substance use [43]. For this RCT, 126 Hispanic families were randomly assigned to either BSFT or group treatment control. BSFT is a family-centered intervention that identifies and redirects maladaptive family interactions in order to improve familial relationships and improve adolescent behavior and substance use. The control consisted of a participatory learning group intervention, involving only the adolescent. Urine toxicology validated self-reported drug use. Results demonstrated that individuals in the BSFT condition experienced greater improvement in parent reports of adolescent behavior, marijuana use, and observer ratings and self-reports of family functioning. From these results, the researchers concluded that brief family therapy was effective in improving behavior and substance use in Hispanic adolescents and can be implemented in accordance with the severity of the adolescent’s condition. This randomized controlled trial offered high-level evidence for the use of BSFT for Hispanic adolescents.

Santisteban et al. evaluated the results of a small RCT to investigate the relative efficacy of a culturally informed family-based intervention for Hispanic adolescents with substance-use disorder, as compared to traditional family therapy (TFT), in reducing adolescent drug use and behavioral problems [44]. The family-based intervention was Culturally Informed and Flexible Family-Based Treatment for Adolescents (CIFFTA), with foundations in structural family therapy. CIFFTA is a culturally adapted, family-focused intervention that combines structured family therapy with adolescent interventions and psychoeducation. Both CIFFTA and TFT are structured forms of family therapy, but CIFFTA integrates individual and cultural components to address topics such as acculturation. Results were measured via the Revised Behavior Problems Checklist and the Youth Self-Report; drug use was assessed using timeline follow-back over a 31-day period and verified via urine toxicology. The study consisted of 28 Hispanic adults and their families. Researchers found that CIFFTA participants showed a greater reduction in drug use than individuals in TFT. However, there were no significant effects on parent-reported behavior problems. From this study, researchers concluded that CIFFTA is more effective in reducing drug use in this population compared to traditional family therapy. This study offered encouraging evidence for use of CIFFTA despite a relatively small sample size (n=28).

Tucker et al. conducted a pilot RCT to improve nicotine patch adherence in Latino patients with HIV/AIDS [45]. In the study, 40 patients were randomized to a control or intervention condition. The control condition consisted of a standard 5 A’s smoking cessation counseling session and eight weeks of nicotine patch treatment. The intervention condition consisted of a similar course of treatment with an additional 10-min module focused on improving nicotine patch adherence. Results were measured via seven-day point prevalence and 90-day continuous abstinence, confirmed via carbon monoxide and self-report of days of patch use. Abstinence rates in the intervention condition were two to three times larger than the control group, though these results were not statistically significant. Similarly, nicotine patch adherence was greater for the intervention group; however, these results did not reach statistical significance. The researchers concluded that an additional counseling module may be helpful in improving smoking cessation and nicotine patch use. This randomized controlled trial presented low-level evidence for the value of the intervention module.

Telepsychiatric therapy interventions: Paris et al. conducted an RCT to evaluate the efficacy of adding a culturally adapted version of web-based cognitive behavioral therapy, computer-based training for cognitive behavioral therapy (CBT4CBT), to standard outpatient psychiatric or addiction treatment (TAU) to improve substance use outcomes, as compared to standard outpatient mental health and addiction treatment [46]. The CBT4CBT intervention was a seven-session online program that taught patients coping skills via videos, exercises, and culturally adapted scenarios in order to reduce substance use. The study consisted of 92 Latino individuals with Spanish as their principal language. Patients were evaluated via available Spanish-language assessments, such as the Structured Clinical Interview for DSM-IV, the Addiction Severity Index, and day-by-day self-reports of drug and alcohol use. Self-reports were validated via urine toxicology. The TAU+CBT4CBT group demonstrated a significantly greater reduction in frequency of primary substance use over time, as compared to the TAU group. From these results, the research group concluded that web-based psychotherapy interventions for this population are effective in reducing substance use. This randomized controlled trial presented high-level evidence for the utility of CBT4CBT towards addiction treatment of Latino patients.

Part 3: Therapeutic Interventions to Treat Other Disorders in Hispanic/Latinx U.S. Populations

Conventional therapy interventions: There is a paucity of literature on the efficacy of therapeutic interventions in treating mental health disorders in Hispanic/Latinx individuals beyond depression and substance use disorders; perhaps the most studied of these are interventions to reduce symptoms of anxiety (Table 3). For example, La Roche et al. conducted a non-randomized, controlled clinical trial investigating the efficacy of a culturally competent relaxation intervention in improving anxiety and depression severity in 44 Latinx patients [47]. The intervention used culturally adapted guided imagery and relaxation activities to improve anxiety symptoms; participants in the intervention group had statistically significant reductions in anxiety symptoms from the beginning to the end of the program as measured by the Beck Anxiety Inventory on multivariable linear regression (ß = -0.28, p<0.05), and had no statistically significant reductions in depressive symptoms as measured by the Center for Epidemiological Studies Depression Scale (CES-D). The authors concluded that due to the difference in improvement between anxiety and depression, it is necessary to differentiate treatments for these two disorders. With a non-randomized, controlled trial design and a moderate sample size (n=44), this study provided low-level evidence of the intervention's effectiveness for anxiety.

**Table 3:** Psychotherapy trials for disorders other than depression or substance use in Hispanic/Latinx U.S. populations. CTS-2: revised Conflict Tactics Scale; PDS: Post-traumatic Diagnostic Scale; ED: eating disorder; EDE: eating disorder examination; RSE: Rosenberg Self-esteem Scale; CSQ: Client Satisfaction Questionnaire; MCS: Mental Health Composite Summary; ICS: inhaled corticosteroids; PDSS: Panic Disorder Severity Scale; CGI: Clinical Global Impression Scale; MARS: Medication Adherence Report Scale; ACQ: Asthma Control Questionnaire; CBPT: cognitive behavioral psychophysiological therapy; MRT: music and relaxation therapy; SCL-90-R: Anxiety subscale of the Symptom Checklist-90-R; RCMAS: Revised Children's Manifest Anxiety Scale; RCMAS/P: RCMAS parent; SMAS: Stephenson Multigroup Acculturation Scale; DSI: Dominant Society Immersion Subscale; ESI: Ethnic Society Immersion; CAPS: Clinician-Administered PTSD Scale; CGI-SD: Clinician Global Impression of Change Scale; SCID-I/P: Structured Clinical Interview for DSM-IV Axis I Disorders; MOS/RAND-36: RAND-Medical Outcomes Scale 36-item health survey; LIFE-Psychosocial: Psychosocial Functioning Scale of the Longitudinal Interval Follow-Up Evaluation; PTCI: Post-traumatic Cognitions Inventory; FSSC-R: Fear Survey Schedule for Children-Revised; CBCL: Child Behavior Checklist; CBT-BN+FE: CBT for bulimia nervosa with family enhanced modules; CIFFTA: Culturally Informed and Flexible Family Treatment for Adolescents; ITAU: Individually Oriented Treatment-As-Usual; CBCL: Child Behavior Checklist; FES: Family Environment Scale; CES-D: Center for Epidemiological Studies Depression Scale; INDCOL: Individualism-Collectivism Scale; CCRI: Culturally Competent Relaxation Intervention; TF-CBT: trauma-focused cognitive behavioral therapy; CPSS-5: Child PTSD Symptom Scale for DSM-5; RCADS-SV: Revised Child Anxiety and Depression Scale; CA-CBT: culturally adapted CBT; CBTgsh: cognitive behavioral therapy-based guided self-help

Studies	Study title	Disorder	Study type	Publication year	Data source	Setting	Study, n	Study design	Main outcome	Measure instrument	Main findings
La Roche et al. [47]	A culturally competent relaxation intervention for Latino/as: assessing a culturally specific match model	Multi (anxiety+depression)	Clinical efficacy	2011	Clinical trial	Community Health Center in Boston, MA	44	Participants received a CCRI in a group setting for weekly 1-h sessions for 8 weeks. Outcomes were measured at baseline, post-intervention, and at 3-month follow-up	Anxiety and depression severity	BAI; CES-D; INDCOL	Participants had a significant reduction in the intensity of anxiety symptoms. There were no statistically significant reductions in depression symptoms
Pina et al. [48]	Exposure-based cognitive behavioral treatment for phobic and anxiety disorders: treatment effects and maintenance for Hispanic/Latino relative to European-American youths	Anxiety	Clinical efficacy	2003	Secondary analyses of previous clinical trials	Miami research center	131	Participants of 2 previous clinical trials were examined for treatment response and maintenance to exposure-based CBT	Phobic and anxiety disorder recovery rates and symptoms	RCMAS; RCMAS/P; FSSC-R; CBCL-internalizing and externalizing	There was no statistical significance between the proportion of European-American and Hispanic/Latino youths who had recovered post-treatment. Exposure-based CBT produced positive treatment gains and maintenance for Hispanic/Latino youths
Chavira et al. [49]	Treatment engagement and response to CBT among Latinos with anxiety disorders in primary care	Anxiety	Clinical efficacy	2014	RCT	17 Primary clinics in: Little Rock, AK, LA, and San Diego, CA, and Seattle, WA	336 (85 Latino; 251 non-Latino white)	Patients were randomized to an intervention or usual care condition. Participants in the intervention arms could choose their treatment (CBT, medication, or both).	Clinical improvement and functioning	Anxiety Sensitivity Index, BSI-12, PHQ-8, MCS, SDS	There were no statistically significant differences between Latinos and non-Latino Whites on measures of clinical outcome, including anxiety sensitivity, depression, cognitive and somatic anxiety, and disability at any assessment point
Feldman et al. [50]	Psychological treatment of comorbid asthma and panic disorder in Latino adults: results from a randomized controlled trial	Panic	Comparative effectiveness	2016	RCT	Clinic in Bronx, NY	53 (485 were initially assessed at pre-screening, but only 53 were randomized in the study)	Participants were randomized to a culturally adapted cognitive behavioral psychophysiological therapy intervention or music and relaxation therapy intervention. Outcomes were measured at baseline, mid-treatment, post-treatment, and 3-month follow-up	Panic disorder severity; asthma control; adherence to ICS medication and asthma symptoms/control	PDSS; CGI; Anxiety Sensitivity Index-3; MARS; ACQ	Both groups showed improvements in asthma outcome measures. CBPT showed an advantage over MRT for improvement in adherence to inhaled corticosteroids
Patriarca et al. [51]	Parent involvement enhances CBTs for anxiety disorders in Hispanic/Latino youth: acculturation as a moderator	Anxiety	Comparative effectiveness	2022	RCT	Urban	279 randomized, 211 completed treatment (and used in analysis)	Participants were randomly assigned to one of three arms: individual-youth CBT or parent involvement CBT that targeted either (a) parent-child relationship skills building or (b) parent reinforcement skills	Youth anxiety outcomes	RCMAS; RCMAS/P and youth (RCMAS); SMAS; DSI; ESI	Youth anxiety outcomes were enhanced in both parent involvement CBTs compared with individual-youth CBT
Galano et al. [52]	Post-traumatic stress disorder in Latina women: examining the efficacy of the Moms' Empowerment Program	PTSD	Comparative effectiveness	2017	RCT	Participants were primarily recruited by staff at community centers serving Latina/o and Hispanic families in southeast MI, northern OH, and southern TX	93	Participants were randomized to waitlist control or directly into intervention (Mom's Empowerment Program). Women were interviewed at baseline and post-intervention (10 weeks), at which point waitlist participants were offered participation in the intervention	PTSD symptoms	CTS-2; PDS	Women who participated in the intervention had a significantly greater reduction in PTSD symptoms than women in the waitlist comparison group
Hinton et al. [53]	CA-CBT for Latino women with treatment-resistant PTSD: a pilot study comparing CA-CBT to applied muscle relaxation	PTSD	Comparative effectiveness	2011	RCT	Urban outpatient clinic	24 randomized and split into two groups	Participants were randomized to receive a culturally adapted form of CBT or applied muscle relaxation and were assessed at baseline and at a 12-week follow-up	PTSD symptom severity; anxiety; nervousness; emotion regulation capacity	PTSD checklist; SCL-90-R; Nervios Scale; Emotion Regulation Scale	Patients in the culturally adapted CBT arm improved significantly more than in the applied muscle relaxation intervention
Kelly and Pich [54]	Community-based PTSD treatment for ethnically diverse women who experienced intimate partner violence: a feasibility study	PTSD and MDD	Clinical efficacy and feasibility	2014	Clinical trial	Urban domestic violence services agency in the Northeast US	Enrollment: 27; final sample size: 22	Participants received community-based group psychotherapy for PTSD. Outcomes were measured at baseline and through 6 months post-intervention	Feasibility of intervention, PTSD symptom severity, HRQoL, perceived social support, and self-efficacy	Self-report	Participants had significant reductions in PTSD and MDD, increased self-efficacy, increased quality of life, and increased perceived social support
Vera et al. [55]	A randomized clinical trial of prolonged exposure and applied relaxation for the treatment of Latinos with post-traumatic stress disorder	PTSD	Comparative effectiveness	2022	RCT	PR	98	Participants were randomized to culturally adapted prolonged exposure therapy arm or applied relaxation arm. Intervention ran for 12-15 weekly sessions, lasting 60-90 min. Primary outcome measured at baseline, post-treatment, and at 3-month follow-up	Clinician-rated PTSD symptom severity	Clinician-Administered PTSD Scale for DSM-5	There was no statistically significant difference in PTSD symptom severity between groups. However, both prolonged exposure therapy and applied relaxation groups experienced statistically significant decreases in PTSD symptoms
Orengo-Aguayo et al. [56]	Trauma-focused cognitive behavioral therapy with Puerto Rican youth in a post-disaster context: tailoring, implementation, and program evaluation outcomes	Multi (PTSD+depression+anxiety)	Clinical efficacy	2022	Clinical trial	Community-based mental health clinics, a primary care clinic in PR, and online	56, 36 successfully completed all study components	Fifteen psychologists were trained in TF-CBT. These psychologists then provided TF-CBT to children and adolescents in weekly individual sessions	PTSD, anxiety, and depressive symptoms	CPSS-5; RCADS-SV	Youth participants experienced statistically significant reductions in PTSD, anxiety, and depressive symptoms
Benítez et al. [57]	Cognitive behavioral therapy for PTSD and somatization: an open trial	PTSD	Clinical efficacy	2013	Open trial	Community clinic in South FL	8	Participants received 10-14 sessions of CBT intervention for PTSD and somatization	Depressive symptoms, somatization symptoms, and PTSD severity	CAPS; CGI-SD; SCID-I/P; MOS/RAND-36; LIFE-Psychosocial; BDI; PTCI	The intervention significantly improved PTSD severity score, depression symptoms, and psychosocial and physical functioning variables
Cachelin et al. [58]	Culturally adapted cognitive behavioral guided self-help for binge eating: a feasibility study with Mexican Americans	Eating disorder	Controlled trial	2014	Prospective Trial	Clinics in Los Angeles, CA	31	Participants followed a self-help manual and attended eight guidance/support sessions	Frequency of binge eating, psychological functioning, and weight loss	EDE; BDI-II; BSI; RSE; BMI; CSQ; Program Evaluation Questionnaire	CBTgsh significantly improved participants’ distress level, BMI, ED psychopathology, and self-esteem
Shea et al. [59]	Mexican American women's perspectives on a culturally adapted cognitive behavioral therapy guided self-help program for binge eating	Eating disorder	Clinical efficacy	2016	Qualitative study	Los Angeles, CA	12	Participants were from a larger investigation. Post-treatment focus groups were conducted with Mexican American women who participated in the intervention. The focus groups engaged in discussion	Thoughts and experiences of the participants on eating behavior, body ideals, and self-guided, culturally adapted CBT therapy	Qualitative content analysis of audiotaped sessions	(1) Eating behavior and body ideals are socially and culturally constructed, (2) a multifaceted support system is crucial to Mexican American women’s treatment engagement and success, and (3) the culturally adapted CBTgsh program is feasible and relevant to Mexican American women’s experience, but it can be strengthened with increased family and peer support
Cachelin et al. [60]	Randomized controlled trial of a culturally-adapted program for Latinas with binge eating	Eating disorder	Comparative effectiveness	2019	RCT	Participants were recruited in community health and mental health settings, local organizations, and at an urban university campus in NC	40	Participants were randomized to 2 arms (CBT-based self-help or waitlist). Results were measured at intake and 12-week follow-up	Frequency of binge eating	Objective binge eating episode frequency	CBT-based self-help can improve binge eating and binge eating symptoms in Latinas
Reyes-Rodríguez et al. [61]	Family involvement in eating disorder treatment among Latinas	Eating disorder	Comparative effectiveness	2019	Secondary analysis of one arm of previous RCT	Mental health clinics around Durham, NC	10 Latinas, 10 relatives (total 20)	Participants were randomized to receive CBT-BN+FE or CBT only. Participants were assessed at baseline, week 6, post-treatment, and 3-month follow-up	Thoughts, feelings, and problem-solving process of participants	Qualitative content analysis	Family intervention might serve as a valuable adjunct to conventional treatment by positively influencing social, family, and emotional support for Latinas with eating disorders
Santisteban et al. [62]	Comparing individually-based and family-based treatments for internalizing, externalizing, and family symptoms in Latino youth	Multi CD, ADHD, and MDD and/or high family conflict	Comparative effectiveness	2022	RCT	University community-based training clinic or a university-based family treatment center	200	Participants were randomly assigned to the CIFFTA arm or the ITAU arm. Participants were assessed at baseline and after 16 weeks of treatment	Treatment retention, parent and youth report internalizing and externalizing child symptoms, and family functioning	CBCL; parent and youth reports from the Family Environment Scale; Cohesion scale; Conflict scale	CIFFTA had significantly higher retention than ITAU, and CIFTA had greater reductions in family conflict. Youth in both conditions had similar reduced internalizing and externalizing symptoms
Childs et al. [63]	Showing up is half the battle: the impact of telehealth on psychiatric appointment attendance for hospital-based intensive outpatient services during COVID-19	Not specified	Clinical efficacy	2021	Observational study	Intensive outpatient services from Yale New Haven Hospital; both virtual and in-person	1008 total unique individuals	The study examined differences in attendance of rapidly deployed telepsychiatric services due to COVID versus in-person services by population group, race, insurance, and clinical treatment program	Appointment attendance	Observational data collected from October 01, 2019 to July 31, 2020	Non-Hispanic White youth had increased attendance for telehealth, and no differences were observed for non-Hispanic Black youth. Decreases were found in telehealth attendance for Hispanic/Latinx youth

Pina et al. conducted a secondary analysis of data from two previously completed randomized controlled trials evaluating exposure-based CBT (a treatment for anxiety consisting of repeated gradual exposure and cognitive behavioral techniques to reduce avoidance and anxiety symptoms) efficacy for youth anxiety disorders, comparing treatment outcomes and maintenance between Hispanic/Latino and European American participants [48]. They used data from 191 participants across both trials, 79 European-American and 52 Hispanic/Latino youths, with additional data from one parent of each participant. Researchers compared the original trials’ measures of diagnosis, as determined by the Anxiety Disorders Interview Schedules for Children-C and -P, the youth-completed measures of the Revised Children’s Manifest Anxiety Scale (RCMAS) and Fear Survey Schedule for Children-Revised (FSSC-R), and the parent-completed measures of RCMAS Parent (RCMAS-P) and Child Behavior Checklist (CBCL). Using equivalence testing analyses with a 10% equivalence criterion, researchers found that diagnostic recovery rates of both groups (83.9% of Hispanic/Latinos; 84.2% of European-American youths), post-treatment scores for FSSC-R (mean=117.49 for Hispanic/Latino youth; mean=115.82 for European-American youth), and post-treatment scores for CBCL internalizing (mean=55.21 for Hispanic/Latino youth; mean=​​54.92 for European-American youth) were statistically equivalent. Researchers concluded that empirical evidence for exposure-based CBT demonstrated similar efficacy with the Hispanic/Latino youth as with European-American youth in the trials. This study analyzed data from two well-designed randomized controlled trials with a large, combined sample size. Accounting for demographic limitations, this study presented strong evidence for its conclusions.

Chavira et al. conducted a secondary analysis of a randomized controlled trial to determine the impact of culture/ethnicity (Latino versus non-Latino White {NLW}) on treatment efficacy for patients with anxiety disorders [49]. The original trial randomized patients to either treatment as usual or the Coordinated Anxiety Learning and Management (CALM) intervention. CALM included the option for cognitive behavioral therapy (CBT), medication, or both. The population of interest for the secondary analysis was both Latinos and NLWs in the intervention group, with 85 and 251 participants, respectively. Researchers looked at both engagement outcomes (attendance, homework adherence, understanding of CBT principles, and commitment to treatment) as well as clinical outcomes (the Anxiety Sensitivity Index, the Brief Symptom Inventory {BSI-12}, the PHQ-8, the Mental Health Composite Summary {MCS} from Short Form Scale-12, and the Sheehan Disability Scale {SDS}). While differences in outcomes based on these scales were largely non-significant, there were significant differences found in the number of sessions attended and patients’ understanding of CBT principles, with Latinos attending fewer sessions (t=3.28, p=0.004) as well as receiving lower scores for understanding (t = -2.38, p=0.017). However, ratings of satisfaction with mental healthcare were similar for both ethnic groups at 18 months (t = -0.33, p=0.743). The researchers concluded that their findings show CBT can be effective for Latinos. This secondary analysis of a well-designed and large-sample randomized controlled trial offered high-level support for this conclusion.

Feldman et al. conducted a randomized controlled trial to compare the efficacy of treatment options for Latinos with comorbid asthma and panic disorder (PD) [50]. The comparison was drawn between culturally adapted cognitive behavioral psychophysiological therapy (CBPT) to music and relaxation therapy (MRT). The CBPT intervention combined CBT and education on how to distinguish asthma from panic symptoms; MRT consisted of listening to relaxing music and practicing breathing exercises to improve anxiety and panic symptoms. The participants were 53 Latinos, mainly Puerto Rican and female, and they were randomized to either intervention for eight weeks. Outcomes included measures of PD severity, asthma control, and adherence to inhaled corticosteroids (ICS). Researchers found that both intervention groups showed improvement across outcome measures for PD severity (CBPT, p<0.0001; MRT, p<0.0001) and asthma control; CBPT demonstrated an advantage over MRT for improvement in adherence to ICS, as measured by the Medication Adherence Report Scale (d=1.07, p=0.006). Researchers concluded that both interventions can be effective for treating asthma-PD comorbidity in a high-risk, ethnic minority population, with CBPT possibly offering more benefits for medication adherence. With a moderate sample size, this randomized controlled clinical trial offered good evidence for the use of both interventions.

More recently, in 2022, Patriarca et al. conducted a randomized controlled trial to compare the differential efficacies of individual cognitive behavioral treatment (CBT) to two other parent-involved forms of CBT which either focused on decreasing parent psychological control or decreasing parent use of negative reinforcement for Hispanic/Latino (H/L) youth with anxiety disorders [51]. They were also interested in seeing how parent acculturation and enculturation moderated youth anxiety outcomes after CBT. The study involved 211 H/L youths aged six to 16 years, and they received one of the three treatments for 12-14 weekly sessions. Researchers measured youth anxiety severity as well as acculturation and enculturation for comparison. They found that youth anxiety outcomes improved under both forms of parentally involved CBT (pre-treatment mean=13.21, post-treatment mean=8.31, z=10.96, p<0.001, d=0.94) compared to individual CBT (pre-treatment mean=12.14, post-treatment mean=7.12, z=10.27, p<0.001, d=0.84). Lower levels of parent acculturation were associated with enhanced youth anxiety outcomes in the parentally involved CBT that focused on negative reinforcement. Conversely, at higher levels of parent acculturation, the parentally involved CBT that focused on reducing parental psychological control had enhanced youth anxiety outcomes. Researchers concluded that their study showed the efficacy of CBT in treating anxiety disorders in H/L youth, as well as for creating parentally involved treatments based on parents’ acculturation. Although this clinical trial lacked a control group, its random assignment, large sample size, and substantial duration enabled it to produce promising evidence for the use of parent-involved CBT over individual CBT in H/L youth populations.

Additional trials have focused on studying outcomes in patients experiencing PTSD symptoms. Galano et al. conducted a randomized controlled trial to test the efficacy of a Spanish-language adaptation of the Moms’ Empowerment Program, a 10-week group treatment program for women who have experienced intimate partner violence (IPV); it included education about IPV and help connecting to community resources [52]. The study participants were 93 low-income, mostly immigrant Latinas, who were all Spanish-speaking, had exposure to IPV, and lived with PTSD. Women who were in the intervention had a significantly greater reduction in PTSD symptoms (as measured by the Post-traumatic Diagnostic Scale) than those in the control group (-5.8±10.1 versus 0.2±9.1, p<0.05 on multivariable linear regression). Researchers concluded that the adapted program was able to address several mental health needs for Latinas with experience in IPV, and that this study demonstrated that tailoring current treatment programs for IPV can be done so that it is both effective and culturally sensitive. This well-designed randomized controlled trial produced strong evidence in support of the intervention.

Hinton et al. conducted a randomized controlled trial to compare the clinical efficacy of a culturally adapted form of cognitive behavioral therapy (CA-CBT) for PTSD to applied muscle relaxation (AMR) in 24 female Latino patients with treatment-resistant PTSD [53]. CA-CBT was culturally adapted by incorporating culturally relevant idioms for distress and interpretations of somatic symptoms into standard CBT for PTSD. Meanwhile, the AMR intervention taught patients to systematically tense and relax muscle groups and to use this technique to reduce physical tension and anxiety when needed. The patients received either treatment across 14 weekly sessions. Researchers were interested in measures of PTSD, anxiety, culturally relevant idioms of distress, and emotion regulation ability. Patients in the CA-CBT group were found to improve significantly more than those in the AMR group. Researchers concluded that CA-CBT can be beneficial for treatment-resistant PTSD in Latino women. As a small (n=24) randomized controlled trial, this study presented promising evidence for the use of CA-CBT in Latina patients with treatment-resistant PTSD.

Kelly and Pich conducted a community-based participatory research (CBPR) driven single-arm pilot trial of community-based PTSD treatment for 22 Latinas who had experienced intimate partner violence (IPV) [54]. This trial, conducted in the American Northeast, consisted of six to 10 sessions of weekly psychotherapy groups incorporating supportive therapy (group-based counseling focused on providing emotional support and strengthening self-efficacy), psychoeducation, and self-care strategies; the intervention was modified during the study in response to feedback, adding an acceptance and commitment therapy-based approach (mindfulness and acceptance strategies to help reduce avoidance of distressing trauma-related thoughts and encourage value-aligned behaviors) for a subset of participants. For those completing the six-month intervention (n=15), PCL scores decreased from a baseline of 59.0 to 44.1 (p=0.003). Secondary outcomes, including quality of life and perceived social support measures, also showed promising improvements. From this work, the authors concluded that CBPR was an effective approach to develop and study a group psychotherapy intervention in a domestic violence services agency; their single-center pilot offered promising support for this claim.

Vera et al. conducted a randomized controlled trial comparing a culturally adapted prolonged exposure (PE) intervention to applied relaxation (AR) among Spanish-speaking Latinos with PTSD in Puerto Rico [55]. PE consisted of psychoeducation and repeated exposure to traumatic memories/reminders, and AR included patient education on how to recognize early signs of anxiety and respond using muscle relaxation techniques. They had 98 total participants with 49 in each treatment condition, and participants received treatments for 12-15 weekly sessions. Researchers were primarily interested in clinician-rated PTSD symptom severity, with secondary outcomes of interest being depression and anxiety (measured via the PTSD scale for DSM-5, PHQ, and State-Trait Anxiety Inventory). Study results showed a large within-group effect of treatment on PTSD symptoms (PE: d=1.29, 95% CI: 1.12, 2.05; AR: d=1.38, 95% CI: 1.21, 2.19); however, the between-group effect on PTSD symptoms was not significant. Researchers also observed significant within-group decreases in depression and anxiety symptoms. They conclude that these findings demonstrate the potential benefit of PE and AR as treatments for Spanish-speaking Latinos with PTSD. This well-designed randomized controlled trial strongly supported the authors’ conclusions.

Orengo-Aguayo et al. conducted a clinical trial to evaluate the feasibility of implementation and the effectiveness of trauma-focused cognitive behavioral therapy (TF-CBT) in a Caribbean post-disaster context with Hispanic youth [56]. The participant population included 56 Puerto Rican children and adolescents, aged five to 18 years, referred for treatment of post-traumatic stress symptoms. Outcomes included both PTSD symptoms as reported by the Spanish version of the Child PTSD Symptom Scale for DSM-5, self-report (CPSS-5), and anxiety and depression scores reported by the Spanish form of the Revised Child Anxiety and Depression Scale-short version (RCADS-SV). Researchers found large effect sizes for reductions in youth-reported post-traumatic stress symptoms (Cohen’s d=1.32), depressive symptoms (Cohen's d=1.32), and anxiety symptoms (Cohen’s d=1.18). Researchers conclude that TF-CBT was an effective treatment to address trauma-related concerns for youth in Puerto Rico within a disaster context. This non-randomized, controlled clinical trial offered low-level but promising evidence for the effectiveness of TF-CBT for Puerto Rican youth.

Benítez et al. conducted an open trial study to examine the feasibility, acceptability, and treatment outcomes of an adapted cognitive behavioral therapy (CBT) for PTSD and medically unexplained physical symptoms (MUPS) for eight Latino patients, seven of whom were women [57]. There was a significant difference between pre- and post-treatment depression symptoms measured via BDI scores (t_2_{6}=3.91, p=0.008) and psychological and physical functioning measures measured via LIFE-household activities and satisfaction scores (t_2_{7}=2.80, p=0.026 and t_2_{7}=2.36, p=0.050). They observed a small to moderate effect in PTSD severity scores, with 50% of the patients no longer meeting PTSD criteria. Researchers concluded that their study offered preliminary support for the CBT intervention in Latino individuals with PTSD and MUPS. The study’s limitations were consistent with the authors' statements.

Other trials in Latinx populations have focused on eating disorders. Cachelin et al.​ reported a controlled trial to test feasibility and initial efficacy of a culturally adapted cognitive behavioral self-help program to treat binge eating disorder (BED) and related problems in Mexican Americans [58]. Participants were 31 women from Los Angeles who had been diagnosed with binge eating disorder (BED), recurrent binge eating, or bulimia nervosa; participants received treatment for three months. BED taught participants self-monitoring, regular eating, and relapse prevention via eight brief, culturally adapted guidance sessions over 12 weeks. Researchers were interested in binge eating frequency, psychological functioning according to the Brief Symptom Inventory (BSI), body mass index (BMI) weight loss, and depression measured by the Beck Depression Inventory-II (BDI-II). They also used the Rosenberg Self-Esteem (RSE) Scale to measure participant changes in self-esteem. Of the 20 participants who completed the study, 97% attended scheduled support sessions and 92% completed weekly monitoring assignments. Using intention-to-treat analyses, researchers found that 36% of participants were abstinent from binge eating at post-treatment and 39% were in diagnostic remission, with no significant difference between these groups on ANOVA. Further results showed significant improvement from baseline in distress level (p=0.024), BMI (p=0.047), eating disorder psychopathology (eating concern p=0.024; shape concern p=0.013; weight concern p=0.015), and self-esteem (p=0.016). Researchers concluded that the culturally adapted treatment program for binge eating is feasible and efficacious for improving symptoms of binge eating in Mexican American women but acknowledged the lack of a control group. This uncontrolled, small-sample clinical trial presented low-level but promising evidence in support of its intervention to treat eating disorders for Mexican American women.

Shea et al. published a qualitative study done as part of a qualitative investigation into the feasibility and efficacy of a culturally adapted cognitive behavioral therapy-based guided self-help (CBTgsh) for Mexican American women with binge eating disorders [59]. CBTgsh was a 12-week program in which participants followed a manual and attended eight guidance/support sessions to help them establish regular eating patterns, develop alternative behaviors to binge eating, and prevent relapses. They conducted a post-treatment focus group with 12 Mexican American women from the study with bulimia nervosa and binge eating disorder. Researchers found three themes in the data collected. First, they found that eating behavior and body ideals are socially and culturally constructed; second, a multifaceted support system is important for Mexican American women’s treatment engagement and success; third, the culturally adapted CBTgsh program is both feasible and relevant to Mexican American women’s experience. They concluded that their findings provided suggestions for further improvement of CBTgsh for this group. This small sample (n=12), qualitative study offered encouraging evidence for the use of CBTgsh for Mexican American women with binge eating disorders.

Cachelin et al. ​conducted a randomized controlled trial on a culturally adapted treatment program [60]. In this study, researchers examined the efficacy of culturally adapted cognitive behavioral therapy-based guided self-help (CBTgsh) for binge eating in improving symptoms from baseline to a 12-week follow-up. The participants for this study were 40 overweight or obese Latinas with binge eating disorder (BED). Researchers were interested in comparing changes in depression, psychological distress, BMI, and frequency of binge eating, all measured the same as in the above 2014 study. CBTgsh resulted in significant reductions in frequency of binge eating (t{20}=3.18, p=0.005, d=1.0), depressive symptoms (BDI-II total, t{20}=3.50, p=0.002, d=0.48), and psychological distress (BSI total, t{20}=2.36, p=0.029, d=0.50), and notably, 47.6% of participants in the intervention group were abstinent from binge eating at the 12-week follow-up. However, changes in distress regarding binge-eating, eating disorder psychopathology (shape concern), and weight loss were not significant. The reductions in outcomes, as well as abstinence from binge eating, were unique to the treatment group, as the waitlist (control) group showed no significant changes from baseline to the 12-week follow-up (p>0.05). Researchers concluded that CBTgsh can be effective for addressing the needs of Latinas with BED and can improve symptoms. As a randomized controlled trial with a moderate sample size, this study provides good evidence for using CBTgsh for Latinas with BED.

Reyes-Rodríguez et al. conducted a secondary qualitative analysis using data from 10 Latinas with eating disorders and 10 relatives from the Promoviendo una Alimentación Saludable trial, where the Latinas and relatives were randomly assigned to receive six family enhancement sessions, consisting of six sessions of eating-disorder psychoeducation and guidance on how to best support family members with EDs as a caregiver [61]. Researchers were interested in the qualitative content of participants' family enhancement sessions. They report several benefits to having family involvement in treatment of eating disorders (ED), which included patients being able to address stigma and shame by sharing their struggle with family members. Several patients reported that including a family member helped them with treatment retention; patients reported that family support influenced positive changes in eating habits; and, finally, the family sessions made a good environment for patients to share triggers associated with their condition. Researchers concluded that the inclusion of family members can have positive effects on continued treatment engagement. This secondary analysis was limited in scope and sample size, presenting potential evidence in support of this intervention.

Santisteban et al. conducted a randomized controlled trial comparing the efficacy of Culturally Informed and Flexible Family Treatment for Adolescents (CIFFTA) with Individually Oriented Treatment-As-Usual (ITAU) for Latino youth and family [62]. Youth had symptoms of two or more DSM-IV-based disorders such as conduct disorder, ADHD, and MDD, and/or high family conflict. Researchers were interested in each intervention’s ability to retain patients, reduce internalizing and externalizing child symptoms (Child Behavior Checklist), and improve family functioning (Cohesion Scale). The study involved 200 Latino adolescents aged 11-14 years, and they received treatment for 16 weeks. The CIFFTA arm combined culturally adapted family therapy, individual adolescent therapy, motivational interviewing, and psychoeducation. Meanwhile, ITAU consisted primarily of adolescent individual therapy (CBT, interpersonal therapy, etc.) addressing depressive symptoms, ADHD, conduct disorder, and family conflict. Researchers found that CIFFTA (83%) had significantly higher retention than ITAU (71%), and youth within groups demonstrated significant reductions in youth and parent reported externalizing and internalizing behaviors (youth external CIFFTA and ITAU d=0.23, d=0.34, p<0.001/youth internal CIFFTA and ITAU d=0.26, d=0.45/parent external CIFFTA and ITAU d=0.73, d=0.92, p<0.001/parent internal CIFFTA and ITAU d=0.68, d=0.96). Additionally, parents in CIFFTA reported significantly greater reductions in family conflict than ITAU (d=0.38, p=0.025). Researchers concluded that their findings show that both interventions were able to successfully reduce behavior problems. This well-powered and well-designed randomized clinical trial presented strong evidence in support of the authors’ conclusions.

Telepsychiatric therapy interventions: Limited trials in telepsychiatry have been conducted for Latinx patients outside of trials in depression and substance use disorders. The only study identified for these other disorders was performed by Childs et al., who conducted a retrospective observational study comparing in-person versus telehealth attendance for intensive outpatient (IOP) services in adolescents and adults with high clinical acuity psychiatric symptoms who were receiving care at Yale New Haven Psychiatric Hospital; IOP services consisted of group psychotherapy for patients and were delivered via specific diagnosis-based treatment programs [63]. Further analysis was done to observe differences in rates of attendance across age, race, insurance, and type of diagnostic treatment program. The study included 88 Hispanic/Latinx participants in the adult population, and 40 in the adolescent population. The authors found a significant increase in the rate of attendance between in-person and telehealth services for Hispanic/Latinx adults (in-person=52.93%; telehealth=61.49%; p≤0.05). For Hispanic/Latinx adolescents, researchers observed a significant decrease in attendance between in-person and telehealth services (in-person=73.15%; telehealth=59.66%; p≤0.05). Overall, researchers concluded that IOP appointment attendance was significantly higher for telehealth compared to in-person services. However, researchers noted that this observational difference in attendance rates between Hispanic/Latinx adults and adolescents warrants further investigation to elucidate potential barriers to care in subpopulations as telehealth services expand. Additionally, while appointment attendance is important for treatment, further work should be done to understand how telehealth impacts outcomes of treatments for high-risk psychiatric patients. With a retrospective observational design, this study offered useful insights, within the context of the limitations the authors identified above.

Discussion

This narrative review synthesized evidence on the efficacy of psychotherapy and telepsychotherapy interventions for U.S.-based Latinx populations, a demographic that comprises over 20% of the population yet experiences profound disparities in mental healthcare access and use. Historically, the psychiatric and health services research literature has treated the efficacy of psychotherapeutic modalities for ethnic minorities as a secondary concern, frequently relegating racial and ethnic analyses to underpowered, "box-checking" subgroup footnotes rather than the primary focus of investigation. Furthermore, a substantial portion of the existing literature claiming to examine interventions in Latinx populations relies on majority-Latinx samples that fail to meet rigorously defined thresholds or draws from international studies that cannot adequately account for the unique systemic barriers, acculturation stressors, and immigration traumas inherent to the Latinx experience in the U.S. By synthesizing only those studies and subanalyses that maintain a strict threshold of ≥95% U.S. Latinx participants, this review provides a uniquely unconfounded baseline of treatment efficacy. We present notable findings surrounding the landscape of psychotherapeutic (including telepsychotherapeutic) interventions for depressive disorders, substance use disorders, and other psychiatric disorders.

Psychotherapeutic interventions for depressive disorders in Hispanic/Latinx U.S. populations are more studied than interventions for any other examined mental health disorder. Multiple high-quality RCTs have been conducted in the field, showing promising clinical efficacy of techniques such as stepped collaborative care and PST-PC sessions, with additional analyses studying behavioral activation therapy and school-based CBT. High-quality RCTs have also been conducted in teletherapy for Latinx patients with depression, finding merit in webcam-based interventions, ECLA (especially ECLA-T), and specific self-help mobile apps. Exploratory analyses have examined schema therapy, BIPT-A, and behavioral versus cognitive therapy, but further assessment of these studies’ validity is warranted given their reliance on small sample sizes, exclusion of control groups, and omission of qualitative evaluations by patients. Taken together, this body of literature suggests several interventions for Hispanic/Latinx individuals with depressive disorders, many of which have strong evidence for clinical utility.

For substance use disorders in Hispanic/Latinx U.S. populations, high-quality RCTs have been conducted to provide evidence of the efficacy of interventions including MET for alcohol use disorder and culturally tailored CBT interventions (A-CBT) for drug abuse disorders. Additional studies have analyzed the impact of psychoeducational groups, treatment adherence modules, and family-backed strategies like BSFT and CIFFTA, but these findings have been limited by insufficient sample sizes, inadequate cultural tailoring, limited study durations, and the lack of control groups. Only one RCT evaluating telepsychiatric therapy interventions for substance use disorders among this demographic was found, revealing a critical and inequitable gap in current telepsychiatry innovation.

Psychotherapy has shown promise to improve outcomes in several other disorders studied in Hispanic/Latinx U.S. populations, most notably for patients with anxiety disorders, eating disorders, and PTSD. High-quality trials for disorders other than substance use disorders or depression have included work by Pina et al. and Chavira et al. exploring CBT for anxiety disorders, Hinton et al. and Orengo-Aguayo et al. exploring CBT for PTSD, and Cachelin et al. and Shea et al. exploring CBTgsh for binge eating disorders [48,49,53,56,58,59]. Medium- and low-quality studies have been conducted to investigate familial and peer involvement in CBT, especially for binge eating disorder. Telepsychotherapy trials in disorders other than depression and substance use disorders in Hispanic and Latinx populations have been quite limited, with only one retrospective observational study for psychiatrically high-risk adolescents and adults.

Overall, we did not find that cultural adaptation led to superior outcomes compared to non-adapted interventions. Many studies, such as Burrow-Sánchez and Hops, found that culturally adapted interventions outperformed non-adapted treatment, and a few, such as Vera et al., found culturally adapted interventions to be acceptable and effective for Latinx patients [42,55]. However, due to the limited number of comparative effectiveness studies in our review, it is difficult to evaluate whether differences in treatment outcomes were solely due to cultural adaptation.

The findings of this review should be interpreted within the context of several salient limitations. First, as a narrative review, this study synthesized and evaluated the available literature on the efficacy of psychotherapy and telepsychotherapy interventions for mental illness among U.S. Latinx populations but did not involve a formal systematic comparison or quantitative synthesis of findings across studies. Second, the heterogeneity of the Latinx population itself presents a significant challenge. The term "Latinx" encompasses individuals of diverse national origins, immigration experiences, acculturation levels, socioeconomic backgrounds, and linguistic preferences; however, this review was unable to draw nuanced conclusions about specific subpopulations. Third, many included studies had limited methodological rigor, with a preponderance of pre-post designs and comparisons against waitlist or treatment-as-usual controls rather than active comparators or head-to-head comparative effectiveness trials, making it difficult to establish the relative superiority of one therapeutic approach over another. Fourth, the definition and degree of "cultural adaptation" for therapies varied widely across studies, with no standardized framework for what constitutes a culturally adapted intervention. Further research could define this framework more fully. Fifth, our strict study inclusion threshold of ≥95% U.S. Latinx participants may have limited our review to studies with highly specific samples and excluded studies with more diverse populations that included substantial Latinx participation. Such studies may offer useful additional insights into mental healthcare access and use among Latinx populations. Sixth, by restricting our search to peer-reviewed journals published in English, we may have missed insights from international journals published in Spanish. Given that our search criteria also included only U.S.-based studies, the potential impact of this limitation may be minimal. Seventh, we conducted the literature search on November 24, 2023, and therefore did not include studies published after this date. Given the continued growth of telepsychotherapy and digital mental health interventions since the COVID-19 pandemic, more recent studies may add to or further refine the findings discussed in this review. Future reviews incorporating more recent evidence, broader population criteria, standardized definitions of cultural adaptation, and more rigorous comparative studies could further clarify the efficacy of psychotherapy and telepsychotherapy interventions among U.S. Latinx populations.

Finally, due to the small number of high-quality clinical trials outside of those for depression and SUDs in the Latinx population, we were unable to perform meaningful meta-analysis on the studies reviewed. Additionally, several reviewed studies had small sample sizes, increasing the likelihood of unstable effect estimates and reduced precision. However, data from these studies are still clinically relevant and provide preliminary evidence of the effectiveness of culturally adapted psychotherapy and teletherapy in the Latinx population.

## Conclusions

Overall, this review paints a picture of a heterogeneous research landscape in psychotherapeutic and telepsychotherapy interventions for Hispanic/Latinx populations within the U.S., with strong clinical evidence for a mere handful of therapies aimed at improving symptoms and outcomes in just a few psychiatric disorders. Our review therefore exposes the stark reality that dedicated, high-quality inquiry into culturally adapted psychotherapy remains sparse, a clear and urgent frontier for future mental healthcare services research. Furthermore, in terms of implications of our findings for clinical practice, we believe that the growing number of studies utilizing telehealth/telepsychotherapy highlights the ability for remote healthcare to reduce health disparities by reducing barriers to care such as transportation and geographic provider shortages. However, in order for telehealth’s full benefit to be realized, disparities in internet access, technological availability, and digital literacy need to be adequately addressed by healthcare systems and policymakers. However, more research is needed to determine how best to serve Latinx patients, a population with a high need for, but an often-limited access to, psychiatric care. Looking toward future research, we believe that there is a need for more comparative effectiveness trials in order to better determine the role of cultural adaptation in treatment outcomes. Additionally, future analyses should include adequately powered RCTs and a standard to compare different cultural adaptation methods; these steps would strengthen an analysis of the role of cultural adaptation in psychotherapy/psychiatric interventions.

## Appendices

**Table 4:** Search strategy, including search criteria, logic, and keywords used for the literature search.

Databases	Strategy
MEDLINE (Ovid)	("Hispanic or Latino"/ or "Hispanic or Latino".mp.) and (Psychotherapy/ or Psychotherapeutic Processes/ or Telepsychiatry/ or Mental Health App/ or Digital Mental Health/)
Embase	hispanic':ab,ti AND (psychotherapy OR telepsychiatry OR 'digital mental health' OR 'digital health app' OR 'psychotherapeutic processes')
PubMed	(telepsychiatry OR mental health app OR digital mental health) AND (hispanic OR latino OR latinx)
PubMed	Psychotherapy [Mesh] AND (hispanic OR latino OR latinx)
PubMed	(("Psychotherapeutic Processes"[Mesh]) AND (hispanic OR latino OR latinx))
Cochrane Library	(telepsychiatry OR mental health app OR digital mental health) AND (hispanic OR latino OR latinx)
